# Intracellular Ca^2+^ and K^+^ concentration in *Brassica oleracea* leaf induces differential expression of transporter and stress-related genes

**DOI:** 10.1186/s12864-016-2512-x

**Published:** 2016-03-09

**Authors:** Jeongyeo Lee, Jungeun Kim, Jae-Pil Choi, MiYe Lee, Min Keun Kim, Young Han Lee, Yoonkang Hur, Ill-Sup Nou, Sang Un Park, Sung Ran Min, HyeRan Kim

**Affiliations:** Korea Research Institute of Bioscience and Biotechnology, 125 Gwahangno, Yuseong-gu, Daejeon, 305-806 Republic of Korea; Systems and Bioengineering, University of Science and Technology, 217 Gajung-ro, Daejeon, Republic of Korea; Environment-friendly Agriculture Research Division, Gyeongsangnam-do Agricultural Research and Extension Service, Jinju, 660-360 Republic of Korea; Department of Biological Sciences, College of Biological Sciences, Chungnam National University, Daejeon, 305-764 Republic of Korea; Department of Horticulture, Sunchon National University, Jeonnam, 540-742 Republic of Korea; Department of Crop Science, College of Agriculture & Life Sciences, Chungnam National University, Daejeon, 305-764 Republic of Korea

**Keywords:** Calcium ion, Potassium ion, Transcriptome, *Brassica oleracea*

## Abstract

**Background:**

One of the most important members of the genus *Brassica*, cabbage, requires a relatively high level of calcium for normal growth (Plant Cell Environ 7: 397–405, 1984; Plant Physiol 60: 854–856, 1977). Localized Ca^2+^ deficiency in cabbage leaves causes tip-burn, bringing about serious economic losses (Euphytica 9:203–208, 1960; Ann Bot 43:363–372, 1979; Sci Hortic 14:131–138, 1981). Although it has been known that the occurrence of tip-burn is related to Ca^2+^ deficiency, there is limited information on the underlying mechanisms of tip-burn or the relationship between Ca^2+^ and tip-burn incidence. To obtain more information on the genetic control of tip-burn symptoms, we focused on the identification of genes differentially expressed in response to increasing intracellular Ca^2+^ and K^+^ concentrations in *B. oleracea* lines derived from tip-burn susceptible, tip-burn resistant cabbages (*B. oleracea* var. *capitata*), and kale (*B. oleracea* var. *acephala*).

**Results:**

We compared the levels of major macronutrient cations, including Ca^2+^ and K^+^, in three leaf segments, the leaf apex (LA), middle of leaf (LM), and leaf base (LB), of tip-burn susceptible, tip-burn resistant cabbages, and kale. Ca^2+^ and K^+^ concentrations were highest in kale, followed by tip-burn resistant and then tip-burn susceptible cabbages. These cations generally accumulated to a greater extent in the LB than in the LA. Transcriptome analysis identified 58,096 loci as putative non-redundant genes in the three leaf segments of the three *B. oleracea* lines and showed significant changes in expression of 27,876 loci based on Ca^2+^ and K^+^ levels. Among these, 1844 loci were identified as tip-burn related phenotype-specific genes. Tip-burn resistant cabbage and kale-specific genes were largely related to stress and transport activity based on GO annotation. Tip-burn resistant cabbage and kale plants showed phenotypes clearly indicative of heat-shock, freezing, and drought stress tolerance compared to tip-burn susceptible cabbages, demonstrating a correlation between intracellular Ca^2+^ and K^+^ concentrations and tolerance of abiotic stress with differential gene expression. We selected 165 genes that were up- or down-regulated in response to increasing Ca^2+^ and K^+^ concentrations in the three leaf segments of the three plant lines. Gene ontology enrichment analysis indicated that these genes participated in regulatory metabolic processes or stress responses.

**Conclusions:**

Our results indicate that the genes involved in regulatory metabolic processes or stress responses were differentially expressed in response to increasing Ca^2+^ and K^+^ concentrations in the *B. oleracea* leaf. Our transcriptome data and the genes identified may serve as a starting point for understanding the mechanisms underlying essential macronutrient deficiencies in plants, as well as the features of tip-burn in cabbage and other *Brassica* species.

**Electronic supplementary material:**

The online version of this article (doi:10.1186/s12864-016-2512-x) contains supplementary material, which is available to authorized users.

## Background

Ca^2+^ and K^+^ are essential macronutrients and normally the two most abundant cations in plants. Ca^2+^ plays a number of roles in stabilizing cell walls and membranes, and as a second messenger [1, 2, 6]. Ca^2+^ is a key component in the signaling of developmental and environmental stresses, including cold, heat, drought, salt, UV light, and touch [7–10]. Cytosolic Ca^2+^ levels increase in plant cells in response to abiotic stress, and with this increase, several mechanisms are simultaneously activated by calcium-interacting proteins, such as Ca^2+^-dependent protein kinases, calmodulin, calmodulin-related proteins, calcineurin-like proteins, and calcium-binding EF-hand proteins [11–13]. The number of genes whose expression is known to be modulated by Ca^2+^ transients in plants is limited, and the mechanisms underlying regulation of gene expression by Ca^2+^ signaling are largely unknown. Increased Ca^2+^ concentrations induce the uptake of K^+^ [14]. K^+^ is essential for enzyme activation, protein synthesis, photosynthesis, osmoregulation, stomatal movement, energy transfer, phloem transport, cation-anion balance, and stress resistance [15]. Low K^+^ status induces the synthesis of reactive oxygen species (ROS) and phytohormones, such as auxin, ethylene, and jasmonic acid [16, 17], whereas high K^+^ concentration induces the expression of K^+^ channel proteins and K^+^ transporters, and regulates stomatal conductance and NADPH oxidase activity, thereby reducing ROS production, maintaining membrane stability, and protecting chlorophyll structure in K^+^-sufficient plants under abiotic stresses [18].

The species *Brassica oleracea* is generally considered to include seven varieties with different morphological characteristics; these are cabbage (*B. oleracea* var. *capitata*), kale (*B. oleracea* var. *acephala*), broccoli (*B. oleracea* var. *italica*), Chinese broccoli (*B. oleracea* var. *alboglabra*), cauliflower (*B. oleracea* var. *botrytis*), Brussels sprout (*B. oleracea* var. *gemmifera*), and kohlrabi (*B. oleracea* var. *gongylodes*). Cabbage is the most economically important member of the genus *Brassica* and contains functional phytochemicals, such as phenolics, vitamins, and minerals, as well as glucosinolates [19], and requires a relatively high concentration of calcium for normal growth [20]. Localized Ca^2+^ deficiency in cabbage leaves causes tip-burn, which is necrosis at the margins of leaves, bringing about serious economic losses [3–5]. Although it has been known that the occurrence of tip-burn is related to Ca^2+^ deficiency [21, 22], there is limited information on the mechanisms of tip-burn or the relationship between Ca^2+^ levels and tip-burn incidence.

Transcriptome analysis provides an efficient means of constructing total expression catalogs, even in the absence of reference sequences, and of analyzing the relative abundance of individual RNAs [23]. Application of transcriptome analysis to gene expression profiling consequently resolved the transcriptional complexity of whole plants and specific tissues under specific environmental conditions [24, 25]. Currently available transcriptome data for *B. oleracea* is not abundant to study the mechanisms underlying plant deficiencies in essential macronutrients. Most studies of the *B. oleracea* transcriptome have focused on genotype [26, 27], tissue- [28–30], or stress-specific [31] gene identification. The mitochondrial transcriptome and microRNAs (miRNAs) of *B. oleracea* have been reported, demonstrating mitochondrial genome evolution and an essential role of miRNAs in biological processes, respectively [32, 33]. To understand gene expression changes in response to intracellular Ca^2+^ and K^+^ concentrations at the whole-genome level in *B. oleracea*, we compared the transcriptomes of three leaf segments (apex, middle, and base) that show different levels of intracellular Ca^2+^ and K^+^ concentration, from three *B. oleracea* lines. In this study, we focused on the identification of the genes differentially expressed based on intracellular Ca^2+^ and K^+^ concentrations in tip-burn susceptible and tip-burn resistant cabbage and kale. These findings may pave the way for further understanding of features of tip-burn in cabbage, as well as in other *Brassica* species.

## Results and discussion

### Distribution of macronutrient cations in *B. oleracea* leaf

The major macronutrient cations Ca^2+^, Mg^2+^, Na^+^, and K^+^ were previously shown to accumulate preferentially in the leaf base (LB) compared with the leaf apex (LA) of tip-burn susceptible and resistant cabbages under normal conditions [21]. We measured the concentrations of the four cations in the leaves of these two cabbage lines and in kale leaves for comparison (Additional file 1: Figure S1). Ca^2+^, Mg^2+^, Na^+^, and K^+^ accumulated to a significantly greater extent in the LB pieces than in the LA pieces of kale (Additional file 1: Figure S1B); these findings are identical to the earlier findings for cabbage leaves [21]. Levels of Ca^2+^ and K^+^ were generally higher by >1.5- and >3.8-fold, respectively, in LB than in LA pieces; they were also present at higher levels in kale than in the cabbages (Additional file 1: Figure S1B). In contrast, the concentration of Na^+^ was higher in tip-burn susceptible cabbage than in either tip-burn resistant cabbage or kale. This higher level of Na^+^ in tip-burn susceptible cabbage could be explained by the Na^+^/K^+^ antagonism high level of Na^+^ inhibits Ca^2+^ and K^+^ absorption [34]. However, no significant differences in Mg^2+^ content were observed among the cabbages and kale (Additional file 1: Figure S1B).

Cytoplasmic Ca^2+^ levels were measured in kale leaf using a dye that fluoresces under visible light upon binding calcium and compared to our previous data for tip-burn resistant and susceptible cabbages [21]. As expected, cytosolic Ca^2+^ concentration was significantly increased in the epidermal cells of kale LB pieces (Additional file 1: Figure S1C), and cytosolic Ca^2+^ levels were higher in all pieces of the kale than in the corresponding pieces of the two cabbages. As a result, nine groups of leaf segments from tip-burn resistant and susceptible cabbage and kale, with the demonstrated relative levels of epidermal cell cytosolic Ca^2+^ and K^+^, were sampled for transcriptome profiling.

### RNA sequencing and assembly

To analyze the induction of differential gene expression by intracellular Ca^2+^ and K^+^ concentration, we generated cDNA sequences from the LA, LM, and LB pieces of tip-burn susceptible and resistant cabbage and kale. From the nine cDNA libraries, 300 million reads (3.3–4.4 Gbp) were generated (Additional file 2: Table S1). After the removal of low-quality sequences (quality score < 20) and short reads (<25 bp), we selected 264,718,946 reads (79.67 % of raw data), retaining mate-pairs for assembly. A total of 205,046 preliminary contigs were obtained from Velvet assembly with average 7.74 reads. Those were grouped into 154,785 contigs by mate-pair information (Table 1). Average length of the contigs was 1283 bp, and length ranged from 200 to 15,158 bp. These contigs represented 58,096 loci as putative non redundant genes, containing an average of 2.66 isoforms (range, 1–140) (Table 1). The homology search annotated 41,526 (71.48 %) and 43,991 (75.72 %) loci of the putative non redundant genes by aligning them with *Arabidopsis thaliana* and *B. rapa* proteins, respectively (Table 1). These annotated loci covered 90 and 93 %, respectively, of *A. thaliana* and *B. rapa* proteins.

**Table 1:** Results of read assembly of *Brassica oleracea* cabbage and kale transcriptomes

	Loci	Transcripts
Assembly		
Number of sequences	58,096	154,785
Minimum	200	200
Maximum	15,158	15,158
Average	978.25	1283.05
Annotation sequences		
*Arabidopsis thaliana*	41,526 (71.48 %)	124,152 (80.21 %)
*Brassica rapa*	43,991 (75.72 %)	130,376 (84.23 %)
Cover to *Arabidopsis thaliana*	32,006 (90.45 %)	32,095 (90.70 %)
Cover to *Brassica rapa*	38,242 (93.23 %)	38,360 (93.52 %)

### Ca^2+^ and K^+^ concentration-dependent candidate transcriptomes of *B. oleracea* leaves

**Fig. 1:**
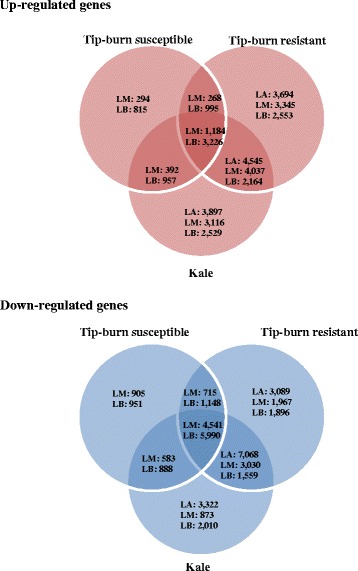
Venn diagram showing numbers of overlapping and nonoverlapping genes differentially expressed by greater than 2-fold in the indicated segments of tip-burn susceptible and resistant cabbage and kale leaves. LA, leaf apex; LM, middle of leaf; LB, leaf base

The Ca^2+^ and K^+^ concentration-dependent changes in transcriptome were measured by comparing the normalized transcriptome data sets with the transcriptome of the LA piece of the tip-burn susceptible cabbage (Additional file 2: Table S1). Loci (27,876) showing significant changes in expression levels were selected based on the two criteria, which “contained ≥ 50 reads with p-value < 0.01”. An absolute value for the transcript level, reflecting an increase or decrease of more than 2-fold, was used for the analysis of differential gene expression. A total of 6128 (10.54 %) loci did not have *Arabidopsis* counterparts, and were considered *B. oleracea*-specific genes with regard to *Arabidopsis* (Additional file 3: Table S2). Among the upregulated genes, a large degree of overlap in expression was observed between tip-burn resistant cabbage and kale. Specifically, 4545, 5221, and 5390 genes expressed in LA, LM, and LB pieces, respectively, overlapped in these two lines (Fig. 1), indicating that genes differentially expressed by more than 2-fold overlapped in the leaf pieces with higher Ca^2+^ and K^+^ contents. A total of 1184 and 3226 genes were up-regulated in the LM and LB pieces, respectively, of all three *B. oleracea* lines. Of the down-regulated genes, 7068, 7571, and 7549 overlapped in the LA, LM, and LB pieces, respectively, of tip-burn resistant cabbage and kale. A total of 4541 and 5990 genes were down-regulated in the LM and LB pieces, respectively, of all three lines (Fig. 1). Shared overexpression of these genes might be related to differences in the intracellular Ca^2+^ and K^+^ concentrations of the different regions of the leaves of *B. oleracea*.

### Tip-burn related phenotype-specific genes expressed in *B. oleracea* lines

**Fig. 2:**
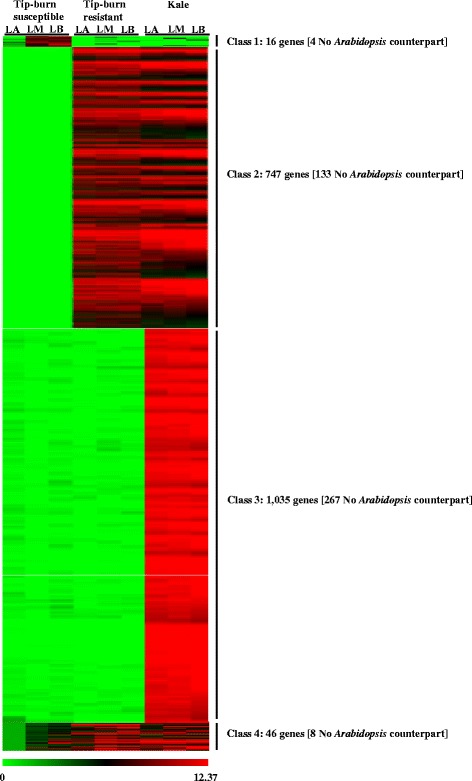
Hierarchical cluster display of tip-burn related phenotype-specific genes. The color scale bar at the bottom of the figure indicates the maximum log_2_ values of selected genes. See also Additional file 5: Table S3. Class 1, specific for tip burn-susceptible phenotype; class 2, up-regulated in tip-burn resistant cabbage and kale; class 3, specific for kale; class 4, up- or down-regulated in all leaf segments compared to LA of tip burn-susceptible cabbage

Hierarchical cluster analysis was carried out on 1844 loci identified as phenotype-specific genes expressed in *B. oleracea* (Fig. 2). Phenotype-specific genes were defined as genes with |log_2_Ratio| ≥ 2 in at least one of the groups, and accompanied by a |log_2_Ratio| < 2 in the other groups. A total of 16 genes, including 4 with no *Arabidopsis* homolog (NA) were specifically expressed in LM and LB pieces of the tip-burn susceptible cabbages (class 1). Together, 747 genes, including 133 NA genes were expressed exclusively in tip-burn resistant cabbage and kale (class 2), indicating that these genes could be related to the trait of tip-burn resistance. The total number of kale-specific genes (class 3) was 1035, and 46 genes had transcript levels that were increased or decreased by more than 2-fold in all tissues (class 4) relative to tip-burn susceptible LA, with 8 NA (Fig. 2).

The 1844 phenotype-specific genes were largely related to stress and transport activity, based on GO annotation (Additional file 4: Figure S2, Additional file 5: Table S3). In the gene ontology (GO) cellular component, most class 1 genes were associated with the nucleus (57.1 %), Golgi apparatus (14.3 %), and cytosol (14.3 %) (Additional file 4: Figure S2), whereas the main cellular components associated with class 2 and 3 genes were nucleus (28.2 and 27.5 %, respectively), chloroplast (13.5 and 13.2 %, respectively), and plasma membrane (13.6 and 12.9 %, respectively). The dominant components for class 4 genes were the nucleus, plasma membrane, and mitochondria, accounting for 33.3, 14.3, and 11.9 % of genes, respectively. In the ontology biological process, genes associated with the developmental processes (13.6 %), response to various stimuli (13.6 %), and transcription (13.6 %) were prominently represented in class 1. Classes 2 and 3 were enriched in genes associated with response to stress (10.4 and 11.6 %, respectively), transport (9.3 and 8.3 %), and protein metabolism (12.5 and 12.3 %, respectively), suggesting that these genes are needed in the presence of higher concentrations of intracellular Ca^2+^ and K^+^. Moreover, the transcriptional response to intracellular Ca^2+^ and K^+^ concentration was similar in cabbage and kale, showing the expression of genes involved in major biological processes and molecular functions. Class 4 was enriched in various genes associated with plant developmental processes and stimulus response, represented by GO terms cell organization and biogenesis (12.9 %), protein metabolism (12.9 %), and response to various stimuli (9.7 %) in the biological process ontology. Among the GO molecular function terms, the main functional groups in class 1 were associated with DNA or RNA binding (16.7 %), protein binding (16.7 %), and transcription factor activity (16.7 %). Classes 2 and 3 showed identical levels of GO term enrichment, i.e., nucleotide binding (14.5 and 14.7 %, respectively), hydrolase activity (11.4 and 10.9 %, respectively), and transferase activity (11.2 and 12.1 %, respectively). The molecular functions DNA or RNA binding (15.2 %), protein binding (10.9 %), and transferase activity (10.9 %) were represented in class 4 (Additional file 4: Figure S2).

Based on the results of GO annotation of phenotype-specific genes, higher Ca^2+^ and K^+^ concentrations were relevant to the expression of transport activity genes or response to stress genes (Table 2). Only one transport activity gene, with GO term “Ca^2+^ activated outward rectifying K^+^ channel protein”, was detected in class 1. The transporter category was composed of 39 and 24 genes in class 2 and class 3, respectively. A number of the transporters were identified in those groups, such as intracellular protein transporter, MATE efflux family protein, sugar transporter, K^+^ transporter, vacuolar protein sorting-related transporter, ABC transporter, ATP/ADP transporter, and other ion transporters. In summary, the class 2 and 3 genes were specialized for the expression of transporter genes and genes involved in ion signaling, compared to class 1. Nine transporters were detected in class 4, including intracellular protein transporter, ABC transporter, lipid transporter, phosphate transporter, phosphatidylinositol transfer protein, and Mg^2+^ transporter (Table 2). The response to stress genes were classified into nine GO categories (Table 2). It is noteworthy that the 19 stress-related genes, including those encoding 6 heat-shock stress proteins, 4 disease-resistance proteins, 3 osmotic stress proteins, 2 light stress proteins, 1 cold-stress and salt-responsive protein, and 1 photoxidative stress-related protein were included in class 2. In class 3, 26 genes related to response to stress were up-regulated, including genes shared with class 2, and additional genes encoding 1 drought stress-related protein and 1 cold-stress-related protein. However, class 4 contained 5 genes encoding disease-resistance proteins, 2 encoding abiotic stress-related proteins, and 1 encoding heat-shock stress-related protein (Table 2). In order to correlate these results with phenotype, we exposed tip-burn susceptible and resistant cabbage and kale plants to temperature stresses and drought stress in two cabbages. Tip-burn resistant cabbage and kale plants showed phenotypes that clearly indicated heat-shock, freezing, and drought stress tolerance compared to tip-burn susceptible cabbage plants (Additional file 6: Figure S3A). Based on these results, we predicted that there is a correlation between intracellular Ca^2+^ and K^+^ concentrations and tolerance of abiotic stress. Basal transcript levels of genes previously identified as abiotic stress-response genes were higher in kale than in tip-burn susceptible and resistant cabbages, and transcript levels were co-regulated in response to abiotic stress, as expected (Additional file 6: Figure S3B). Three (locus_21191, locus_51499) of the tested genes showed lower basal transcriptome level in tip-burn resistant cabbage than in susceptible cabbage. However, the transcriptions of the locus_21191 and locus_51499 in tip-burn resistant cabbage were more increased by the drought or freezing stresses than in susceptible cabbage (Additional file 6: Figure S3B). Consequently, the expression of the locus_22894 was expected to increase more by other stresses in same manner. Our data show that intracellular Ca^2+^ and K^+^ concentrations affected plant responses to environmental stresses by differential regulation of appropriate stress-induced genes.

**Table 2:** Functional categories of phenotype-specific transport activity and response to stress genes expressed in tip-burn susceptible and resistant cabbages and kale

Functional category	Class 1	Class 2	Class 3	Class 4
Transport activity				
Ca^2+^ activated outward rectifying K^+^ channel	1	1		
Intracellular protein transport		11	5	4
ABC transporter		2	1	1
Lipid transporter		1	1	1
Phosphate transporter		1	1	1
MATE efflux family		4	3	
Sugar transporter		3	1	
K^+^ transporter		3	1	
Vacuolar protein sorting-related		2	1	
Vesicle transport protein		1	1	
Nodulin MtN21/EamA-like transporter		1	2	
Aluminium activated malate transporter		1		
Cation/H^+^ exchanger		1		
ATP/ADP transporter		2		
Iron-regulated transporter		1		
Heavy metal transporter		1		
UDP-galactose transporter		1		
Urea transmembrane transporter		1		
Zinc ion transporter		1		
Anion channel			1	
Calcium ion transporter			1	
Inositol transporter			1	
Manganese tracking factor			1	
Nitrate transmembrane transporter			1	
Nuclear transporter			1	
Peptide transporter			1	
Phosphatidylinositol transfer protein				1
Magnesium transporter				1
Response to stress				
Heat-shock stress related		6	3	1
Disease resistance protein		4	10	5
Abiotic stress related		2	2	2
Osmotic stress		3	4	
Light stress related		2	4	
Cold-stress and salt responsive protein		1	1	
Photoxidative stress related		1		
Drought stress related			1	
Cold-stress related			1	

Class 1, specific for tip-burn susceptible phenotype; class 2, up-regulated in tip-burn resistant cabbage and kale; class 3, specific for kale; class 4, up- or down-regulated in all leaf segments compared to LA of tip burn-susceptible cabbage

### Profiling of gene expression based on increasing intracellular Ca^2+^ and K^+^ concentrations in *B. oleracea*

**Fig. 3:**
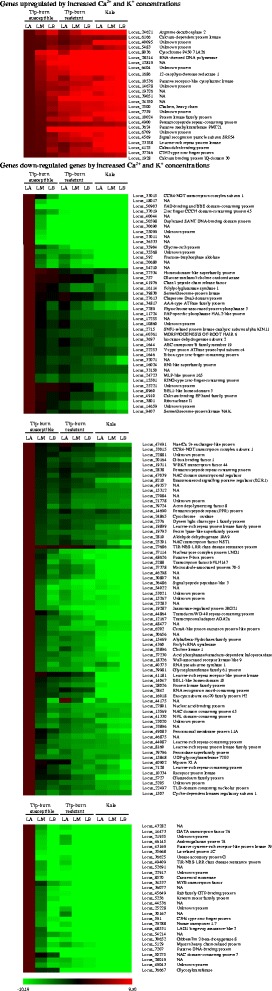
Hierarchical cluster display of genes up- (*n* = 25) and down-regulated (*n* = 140) in Ca^2+^- and K^+^-dependent manner. The color scale bar at the bottom of the figure indicates the maximum log_2_ values of selected genes. See also Additional file 7: Figure S4 and Additional file 8: Table S4

We selected 165 genes that were up- or down-regulated in response to increasing Ca^2+^ and K^+^ concentrations in the three leaf positions of the three *B. oleracea* lines (Fig. 3, Additional file 7: Figure S4). A total of 132 genes, after exclusion of the 33 NA genes, was annotated based on homology to the *Arabidopsis* genes, including 21 unknown genes, with the remainder being specific to the *B. oleracea* genomes. Those genes were clustered hierarchically based on their expression patterns (Fig. 3). Only 25 genes were commonly upregulated in response to increased intracellular Ca^2+^ and K^+^ concentrations in the lines as well as in the leaf positions. Locus_6709 (encoding an unknown protein) was up-regulated to the greatest extent, showing the largest fold change (Additional file 8: Table S4), and was also the most highly expressed gene in the leaf position with the highest concentrations of Ca^2+^ and K^+^, i.e., kale LB. A total of 140 genes were down-regulated in response to increasing Ca^2+^ and K^+^ concentrations, and locus_35568 (encoding an unknown protein) was the gene down regulated to the greatest extent in kale LB. Locus_33015 gene (CCR4-NOT transcription complex subunit 1 domain protein) was the gene most down regulated in all three leaf positions of tip-burn resistant cabbage compared to the susceptible cabbage (Additional file 8: Table S4).

**Fig. 4:**
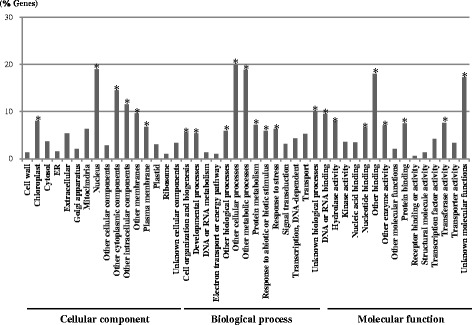
Distribution of gene ontologies cellular component, biological process, and molecular function for the genes up- or down-regulated in response to increasing Ca^2+^ and K^+^ concentrations in the leaf segments of tip-burn susceptible and resistant cabbages and kale plants. *Asterisks* indicate significantly enriched GO terms were adjusted to *P* ≤ 0.001

The selected up- or down-regulated genes were classified according to the GOs cellular component, biological process, and molecular function. A total of 432 GO IDs were obtained for the 132 genes (Fig. 4, Additional file 9: Table S5). In the GO cellular component, the terms “nucleus”, “plasma membrane”, and “chloroplast” were enriched. The terms “cell organization and biogenesis”, “developmental processes”, “protein metabolism”, “response to abiotic or biotic stimulus”, “response to stress”, and “unknown biological processes” occurred most frequently in the ontology biological process. In the molecular function ontology, the terms “hydrolase activity”, “transferase activity”, “unknown molecular functions”, and various “binding” were enriched, accounting for 56.4 % of genes in this ontology (Fig. 4). GO analysis showed that genes associated with the terms “unknown biological processes” and “unknown molecular functions” were enriched, accounting for 10.2 and 17.3 % of genes, respectively (Fig. 4). Based on the GO annotation, those selected genes were considered to be involved in regulatory metabolic processes or stress responses. The data obtained in this study will be informative not only for research on essential macronutrient concentration-related gene expression in *B. oleracea*, but will also be useful for the investigations of Ca^2+^ deficiency disorders in related species.

## Conclusions

The major macronutrient cations Ca^2+^ and K^+^ showed significant preferential accumulation in LB, compared to LA, in tip-burn susceptible and resistant cabbage and kale. Concentrations of Ca^2+^ and K^+^ were more than 2-fold higher in both tip-burn resistant cabbage and kale than in tip-burn susceptible cabbage. Analysis of changes in the transcriptomes of these plants in response to changes in Ca^2+^ and K^+^ levels identified 27,876 loci showing significant changes in the expression level. Among these, 1844 loci were identified as tip-burn related phenotype-specific genes. Of these, 16 were specific for the tip burn-susceptible phenotype; 747 were specific for tip-burn resistant; 1035 were specific for kale; and 46 were up- or down-regulated in all leaf positions compared to the LA of tip-burn susceptible cabbage. The genes specific for tip-burn resistant and kale were largely related to stress and transport activity based on GO annotation. Tip-burn resistant cabbage and kale plants showed phenotypes that clearly indicated heat-shock, freezing, and drought stress tolerance compared to tip burn-susceptible cabbages, demonstrating the correlation between intracellular Ca^2+^ and K^+^ concentrations and tolerance of abiotic stress with differential gene expression. We selected 165 up- or down regulated in response to increasing Ca^2+^ and K^+^ concentrations in three leaf segments of the three *B. oleracea* lines. Based on the GO enrichment analysis, the selected genes were identified as participating in the regulatory metabolic processes or stress responses. This study provides information on the mechanisms of tip-burn and the relationships between intracellular Ca^2+^ concentration and tip-burn incidence in *B. oleracea*, which will be useful for research on Ca^2+^ deficiency disorders in other plant cultivars.

## Methods

### Plant materials and cation measurements

*B. oleracea* plants, tip-burn susceptible (DH line HRIGRU009386, Warwick Crop Centre, UK) and resistant (inbred line No107140, Samsung Seed Co., Korea) cabbages, and kale (inbred line FB, Asia Seed Co., Korea) were grown under controlled conditions in a greenhouse for 90 days at 22 °C with a photon flux density of 100 μmolm^−2^s^−1^ and a 16 h light/8 h dark photoperiod, as previously described [21]. After beginning of the heading process in tip-burn susceptible cabbage, middle leaves (leaf length 8–10 cm) were harvested from five independent leaves from three different plants of two cabbages and kale. The soil used in this experiment was commercial compost soil (Punong, Korea) with an original pH of 6.0 which consist of zeolite, vermiculite, perlite, and coir dust. Available nutrients of the soil were (kg^−1^ soil) 8.7 mg P, 112 mg K, and 170 mg Mg. Determination of the concentration of Ca^2+^ in the kale leaf epidermal cells using Fluo-4/AM ester and ion chromatographic analysis were performed as described before [21]. Two hours after the incubation with Fluo-4/AM ester, cells were examined by confocal microscopy.

### Stress-tolerance assays

To test cold, heat-shock, and drought stress, we prepared seedlings from seven-week-old *B. oleracea* plants. For heat-shock stress treatment, tip-burn susceptible and resistant cabbages and kale plants were exposed to 45 °C for 5 h in the dark. Freezing-stress treatment consisted of exposing the plants to −4 °C for 6 h in the dark, followed by 3 days of recovery under normal growth conditions. For drought stress treatment, soil-grown plants were fully watered, and then irrigation was withheld for 10 days, followed by rewatering. Plant survival was assessed 1 week after rewatering. All experiments were repeated at least twice.

### Library preparation and transcriptome sequencing

We constructed sequence libraries from three segments (LA, LM, and LB) of leaves generated from tip-burn susceptible and resistant lines and kale (Additional file 1: Figure S1A). Total RNA was extracted from 100 mg of each tissue using Trizol reagent (Life Technologies, Carlsbad, CA, USA) according to the manufacturer’s instructions. To remove any DNA contamination, samples were treated using the Oligotex Direct mRNA Mini Kit (Qiagen, Hilden, Germany). The concentration of the mRNA was determined using a Qubit analyzer (Invitrogen, Carlsbad, CA, USA). The cDNA libraries were prepared according to the instructions of the manufacturer of the sequencing system (Illumina, San Diego, CA, USA). Poly(A) mRNA was purified using Sera-Mag Magnetic Oligo (dT) Beads (GE Healthcare, Little Chalfont, Buckinghamshire, United Kingdom) from 20 μg total RNA. The cDNA library products were sequenced on a paired-end flow cell using an Illumina HiSeq™ 2000 system.

### Transcriptome data processing and sequence assembly

To obtain high-quality reads, we trimmed reads with quality scores less than 20 and obtained reads longer than 25 bp in length using SolexaQA (ver. 1.13) software [35]. We used preserved read pairs for assembly by eliminating loss-of-mate read pairs during the preprocessing. We assembled preliminarily sequences using Velvet (ver. 1.2.06) [36] with parameter (K-mer = 57, 59 bp), and then fragmented contigs were revised using the Oases algorithm (ver. 0.2.08) [37]. Oases clustered all possible sequences generated from the same locus, including isoforms. The representative sequence (locus) was defined as the longest sequence among the clustered transcripts.

### Gene annotation and GO categorization

Protein sequences of *B. rapa* and *Arabidopsis thaliana* were downloaded from the *Brassica* database (ftp://brassicadb.org/v1.2/) and the *Arabidopsis* Information Resource (ftp://ftp.arabidopsis.org/home/tair/Sequences/blast_datasets/TAIR10_blastsets/), respectively. To determine the level of sequence conservation, we implemented BLASTx against those two databases with an expected value < 1e^−10^.

GO terms for *A. thaliana* proteins were downloaded from the TAIR website (ftp://ftp.arabidopsis.org/home/tair/Ontologies/Gene_Ontology/). GO terms for the phenotype-specific genes and genes differentially expressed based on increasing Ca^2+^ and K^+^ concentrations were assigned based on their *A. thaliana* syntenic counterparts. GO enrichments were analyzed using Fisher’s exact test (in the Python module [ver. 0.1.4]), and *p* < 0.001 was applied [38].

### Statistical analysis of transcriptome profile

To monitor expression in each segment of cabbage and kale leaf, we mapped high-quality reads to the assembled unigenes using Bowtie (ver. 1.5) with default option [39]. We summarized the overall expression of a gene based on mapped reads in each library and used loci containing mapped reads longer than 50 bp for further analysis. After normalization, we determined the differentially expressed genes (DEGs) by applying fold change analysis and *t*-test with the DEGSeq package [40]. For the fold change method, we used read counts of LA for tip-burn susceptible cabbage as a reference and converted others to fold change. The false discovery rate (FDR) method was used to determine the threshold p-value through a multiple test. We selected the DEGs meeting criteria of FDR ≤ 0.001 and absolute value of |log_2_Ratio| ≥ 2. We applied the hierarchical cluster analysis to DEGs to construct groups showing similar expression pattern among libraries.

### RNA isolation and quantitative real-time PCR (qPCR)

Plant tissue frozen in liquid nitrogen was ground with a mortar and pestle, and total RNA was isolated from leaves using Trizol reagent according to the manufacturer's instructions. ReverTra Ace qPCR RT Kit (Toyobo, Osaka, Japan) was used to carry out first-strand cDNA synthesis. Reverse transcription (RT) was carried out in a 20-μl reaction mixture containing 1 μg RNA; oligo(dT) 20 primer (10 pmol); 10 mM each of dATP, dCTP, dGTP, and dTTP; 5× RT buffer; 200 units of ReverTra Ace reverse transcriptase; and 10 units of RNase inhibitor. The reaction mixture was incubated at 42 °C for 20 min, and the reaction was stopped by heating at 99 °C for 5 min. To determine the expression patterns of genes, RT-PCR was carried out using gene-specific primers and actin 2 (*ACT2*) transcript level as internal standard. RT-PCR was carried out for 28 cycles of template denaturation at 94 °C for 2 min; primer annealing at 55 °C for 30 s; and elongation at 72 °C for 1 min. PCR products were separated by 1.2 % agarose gel electrophoresis.

## Additional files

Additional file 1: Figure S1. Experimental *Brassica oleracea* (tip-burn susceptible, tip-burn resistant, and kale) leaf and the accumulation of Ca^2+^ in epidermal cells (updated to Lee et al., 2013). (DOCX 744 kb) Additional file 2: Table S1. Summary of read number. (DOCX 29 kb) Additional file 3: Table S2. Expression levels of significant changed genes. (XLSX 2520 kb) Additional file 4: Figure S2. Functional categorization of tip-burn related phenotype-specific expressed genes. (DOCX 103 kb) Additional file 5: Table S3. Expression changes of tip-bun related phenotype-specific genes. (XLSX 179 kb) Additional file 6: Figure S3. Phenotypic appearance (A) and gene qRT-PCR results (B) of two cabbages (tip-burn susceptible and resistant lines) and kale under heat-shock stress, freezing-stress and drought stress conditions. (DOCX 2128 kb) Additional file 7: Figure S4. Selected up- or down-regulated genes based on increasing Ca^2+^ and K^+^ concentration in the three lines with three different leaf positions. (DOCX 169 kb) Additional file 8: Table S4. Transporter genes with significantly altered transcript levels. (DOCX 51 kb) Additional file 9: Table S5. Response to stress genes with significantly altered transcript levels. (DOCX 51 kb)

## Abbreviations

LA: leaf apex
LM: middle of leaf
LB: leaf base
NA: no *Arabidopsis* homolog
ROS: reactive oxygen species
