# Supplementary material for: Intracellular Ca2+ and K+ concentration in Brassica oleracea leaf induces differential expression of transporter and stress-related genes
Source: BMC Genomics. 2016 Mar 9;17:211. doi: 10.1186/s12864-016-2512-x (PMC4784358; doi:10.1186/s12864-016-2512-x)
Supplement: Additional file 1: Figure S1. — Experimental Brassica oleracea (tip-burn susceptible, tip-burn resistant, and kale) leaf and the accumulation of Ca2+ in epidermal cells (updated to Lee et al., 2013). (DOCX 744 kb) [file 12864_2016_2512_MOESM1_ESM.docx]

**A
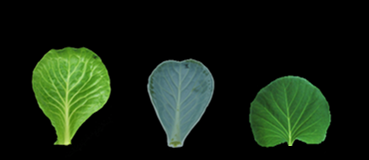
**

**Tip-burn resistant**

**Tip-burn susceptible**

**Kale**

**LA**

**LB**

**LM**

**B**

**10**

**
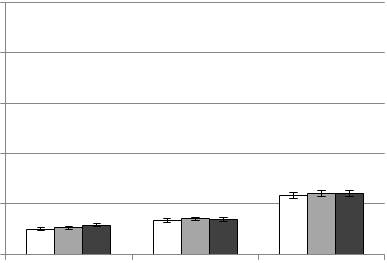
**
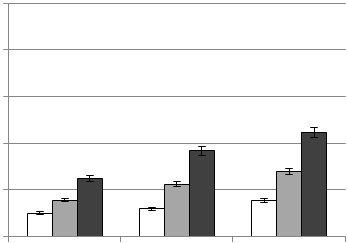


**Ca^2+^**

**Mg^2+^**

**8**

**6**

*

*

*

*

**4**

*

*

**2**

**10**

**0**

**Amount of ion (mg/g dry wt)**


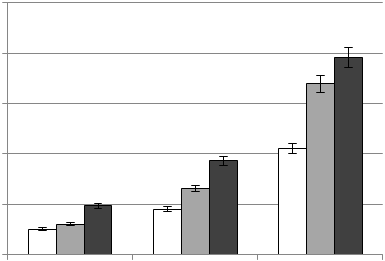

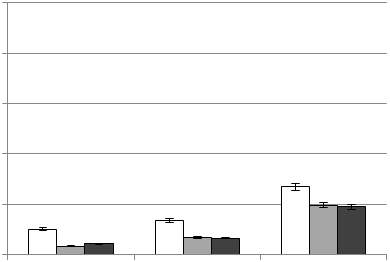


*

**Na^+^**

**K^+^**

**8**

*

**6**

*

**4**

*

*

*

*

**Tip-burn susceptible**

**Tip-burn resistant**


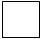

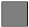

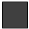


**Kale**

**2**

*

*

*

*

**LA**

**LM**

**LB**

**0**

**LA**

**LM**

**LB**

**Leaf area**

**C**


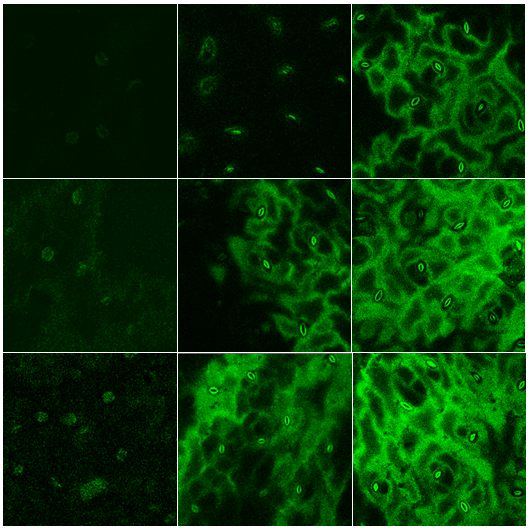


**LA**

**LM**

**LB**

**Tip-burn susceptible**

**Tip-burn resistant**

**Kale**

**Figure S1.** Experimental *Brassica oleracea* (tip-burn susceptible, tip-burn resistant, and kale) leaf and the accumulation of Ca^2+^ in epidermal cells (updated to Lee et al., 2013). **A**, Leaves were divided into three stages and three regions (LA, leaf apex; LM, middle of leaf; LB, leaf base). **B**, Amount of ion in the three regions of *Brassica oleracea*, as determined by an ion pac CS12A. Three 90 days old plants were assayed and three independent experiments were conducted with similar results. Error bars = standard deviation of three independent experiments. *Asterisks* indicate significant differences from the tip-burn susceptible (*P<0.01). **C**, Imaging of cytoplasmic Ca^2+^ within leaf epidermal cells. Samples were incubated with the fluorescent dye, Fluo-4/AM, and monitored by a confocal microscope 2 h later.
