# Supplementary material for: Intracellular Ca2+ and K+ concentration in Brassica oleracea leaf induces differential expression of transporter and stress-related genes
Source: BMC Genomics. 2016 Mar 9;17:211. doi: 10.1186/s12864-016-2512-x (PMC4784358; doi:10.1186/s12864-016-2512-x)
Supplement: Additional file 2: Table S1. — Summary of read number. (DOCX 29 kb) [file 12864_2016_2512_MOESM2_ESM.docx]

**Table S1.** Summary of read number.

|  | **Tip-burn susceptible** | | | **Tip-burn resistant** | | | **Kale** | | |
| --- | --- | --- | --- | --- | --- | --- | --- | --- | --- |
|  | **LA** | **LM** | **LB** | **LA** | **LM** | **LB** | **LA** | **LM** | **LB** |
| **Raw Data** | | | | | | | | | |
| **Read No. ^a^** | 41,185,630 | 32,349,766 | 43,705,284 | 33,718,232 | 37,804,314 | 35,176,096 | 33,609,138 | 38,257,052 | 36,484,142 |
| **Len. (bp) ^b^** | 4,159,748,630 | 3,267,326,366 | 4,414,233,684 | 3,405,541,432 | 3,818,235,714 | 3,552,785,696 | 3,394,522,938 | 3,863,962,252 | 3,684,898,342 |
| **Pre-processing (high-quality sequences)** | | | | | | | | | |
| **Read No. ^a^** | 41,185,630 | 32,349,766 | 43,705,284 | 33,718,232 | 37,804,314 | 35,176,096 | 33,609,138 | 37,257,052 | 36,484,142 |
| **Len. (bp) ^b^** | 3,259,452,130 | 2,612,384,550 | 3,512,934,598 | 2,719,789,764 | 3,043,148,910 | 2,842,755,510 | 2,692,360,987 | 3,059,419,785 | 2,914,104,098 |
|  | 78.36% | 79.95% | 79.58% | 79.86% | 79.70% | 80.01% | 79.31% | 79.18% | 79.08% |
| **Avg. (bp) ^c^** | 79.14 | 80.76 | 80.38 | 80.66 | 80.50 | 80.82 | 80.11 | 79.97 | 79.88 |
| **Paired-end reads** | | | | | | | | | |
| **Read No. ^a^** | 32,186,264 | 25,948,216 | 34,858,212 | 26,997,564 | 30,238,130 | 28,311,464 | 26,746,378 | 30,501,042 | 28,931,676 |
| **Len. (bp) ^b^** | 2,902,701,165 | 2,355,058,981 | 3,160,969,353 | 2,452,222,565 | 2,742,169,369 | 2,567,707,302 | 2,418,876,602 | 2,744,653,258 | 2,613,178,507 |
|  | 69.78% | 72.08% | 71.61% | 72.01% | 71.82% | 72.27% | 71.26% | 17.03% | 70.92% |
| **Avg. (bp) ^c^** | 90.19 | 90.76 | 90.68 | 90.83 | 90.69 | 90.70 | 90.44 | 89.99 | 90.32 |

^a^ Total number reads, ^b^ Total length of sequences, ^c^ An average length of reads.
