# Supplementary material for: Intracellular Ca2+ and K+ concentration in Brassica oleracea leaf induces differential expression of transporter and stress-related genes
Source: BMC Genomics. 2016 Mar 9;17:211. doi: 10.1186/s12864-016-2512-x (PMC4784358; doi:10.1186/s12864-016-2512-x)
Supplement: Additional file 4: Figure S2. — Functional categorization of tip-burn related phenotype-specific expressed genes. (DOCX 103 kb) [file 12864_2016_2512_MOESM4_ESM.docx]

*

*

*

*

*

*

*

*

*

*

*

*

(%)

*

*

*

*

*

*

*

*

*

*

*

*

*

*

*

*

*

*

*

*

*

*

*

*

(%)

*

*

*

Class 1

Class 2


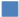

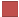

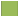

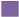


Class 3

*

*

Class 4

*

*

*

*

*

*

*

*

*

*

*

*

(%)

**Figure S2.** Functional categorization of tip-burn related phenotype-specific expressed genes. A threshold of corrected p-value <0.001 and log_2_ >2 was used to judge the significantly enriched GO terms in DEGs. *Asterisks* indicate significant enriched GO terns were adjusted to *P* ≤ 0.001.
