# Supplementary material for: Intracellular Ca2+ and K+ concentration in Brassica oleracea leaf induces differential expression of transporter and stress-related genes
Source: BMC Genomics. 2016 Mar 9;17:211. doi: 10.1186/s12864-016-2512-x (PMC4784358; doi:10.1186/s12864-016-2512-x)
Supplement: Additional file 6: Figure S3. — Phenotypic appearance (A) and gene qRT-PCR results (B) of two cabbages (tip-burn susceptible and resistant lines) and kale under heat-shock stress, freezing-stress and drought stress conditions. (DOCX 2128 kb) [file 12864_2016_2512_MOESM6_ESM.docx]

**Kale**

**Tip-burn**

**resistant**

**Tip-burn**

**susceptible**

**
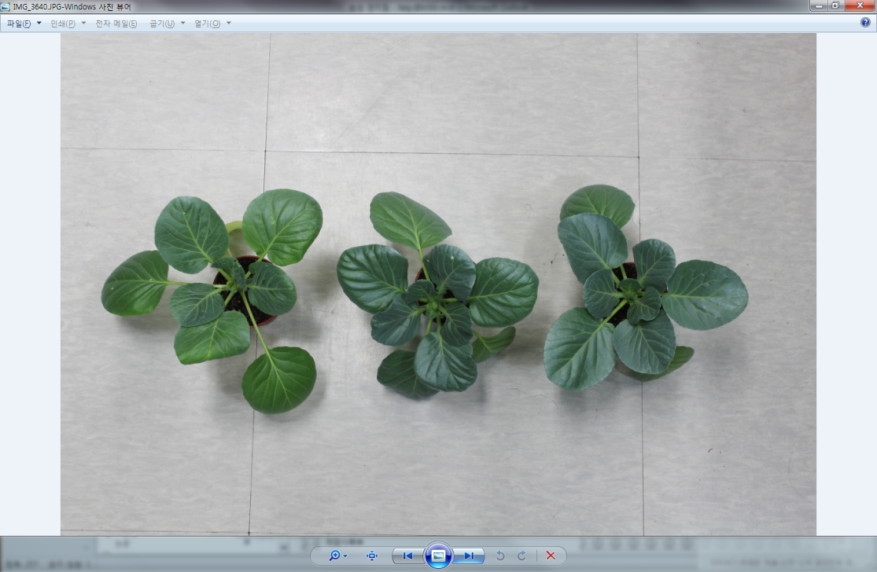
**

**Before treatment**

**
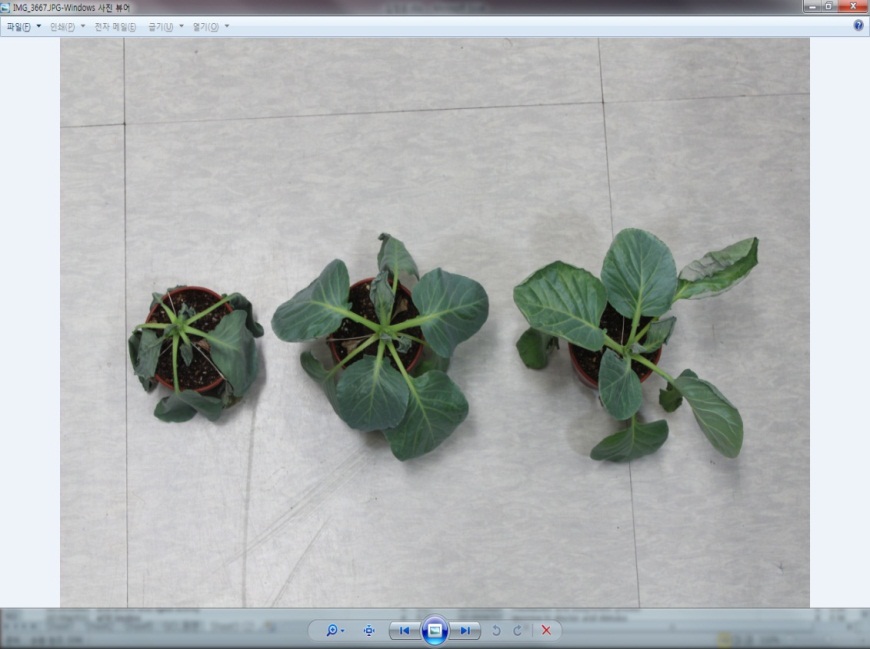
**

**After heat-shock stress**

**
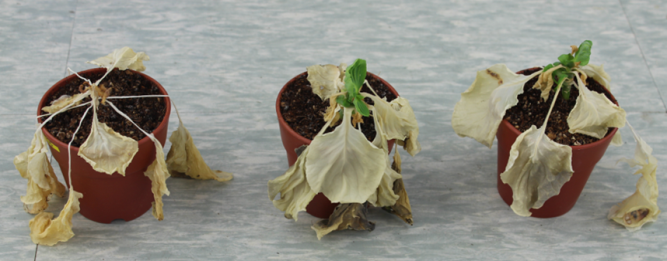
**

**After freezing stress**

**(3 days after recovery)**

**
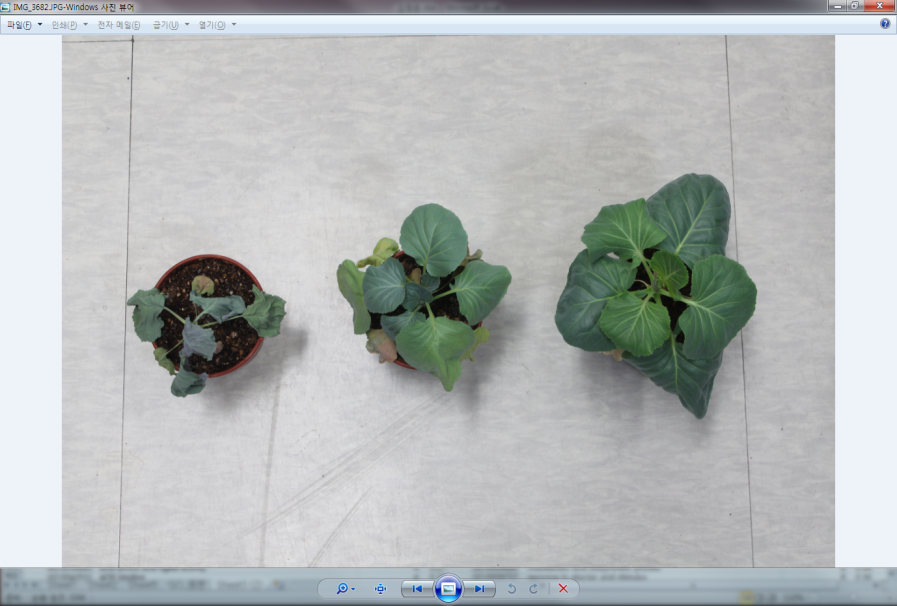
**

**After drought stress**

**(3 days after recovery)**

**B**

**Heat-shock**

**Freezing**

**Drought**


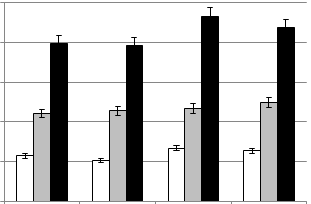

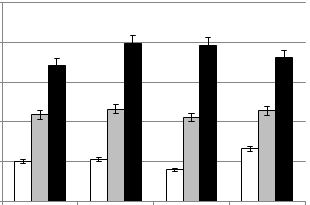

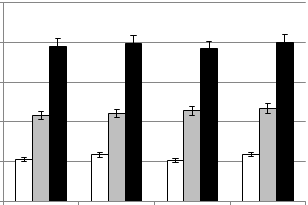


**5**

**4**

**3**

**2**

**1**

**0**

**Locus_9666 (PLAT/LH2 family protein)**

**5**

**4**

**3**

**2**

**1**

**0**


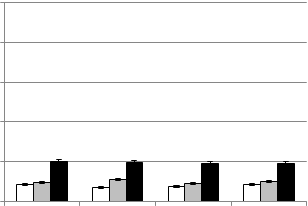

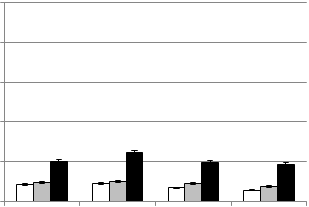

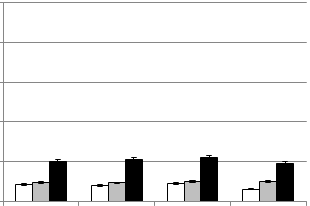


**Tip-burn resistant**


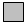


**Tip-burn susceptible**


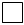


**Locus_15334 (BTB-POZ protein)**

**Kale**


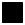


**0 1 3 6 (h)**

**0 1 3 6 (h)**

**0 1 3 6 (h)**

**Heat-shock**

**Freezing**

**Drought**


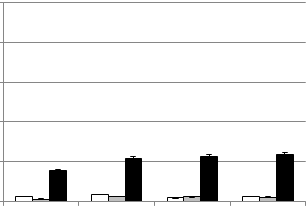

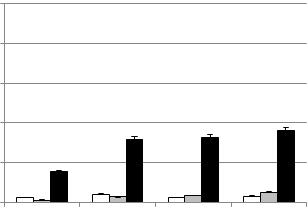

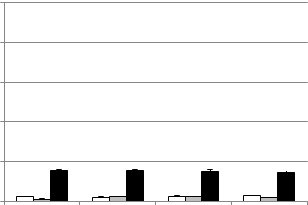


**5**

**4**

**3**

**2**

**1**

**0**

**Locus_22894 (GAST1 protein)**

**5**

**4**

**3**

**2**

**1**

**0**


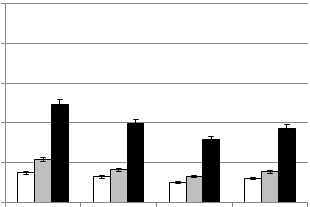

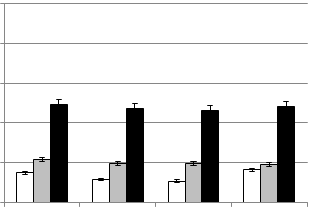

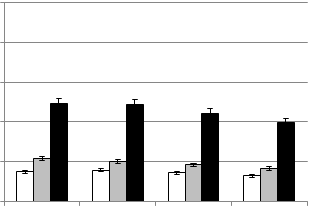


**Locus_1906 (TPR-like protein)**

**5**

**4**

**3**

**2**

**1**

**0**


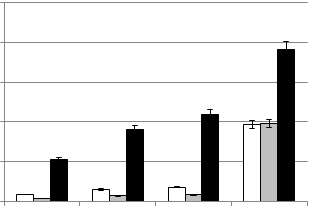

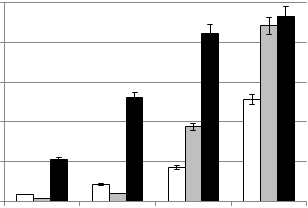

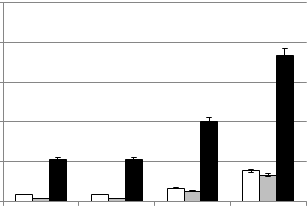


**Locus_21191 (Low temperature and salt responsive protein)**


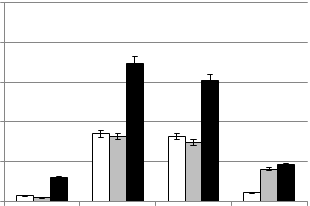


**Locus_51499 (DRE-binding protein 2A)**

**5**

**4**

**3**

**2**

**1**

**0**


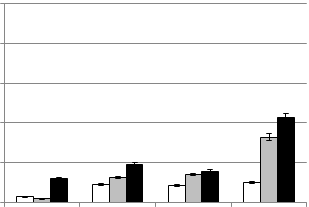

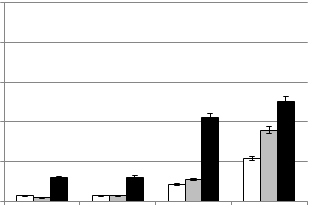


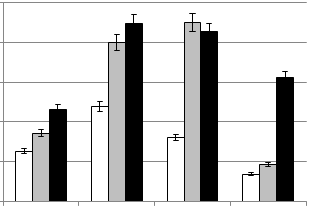

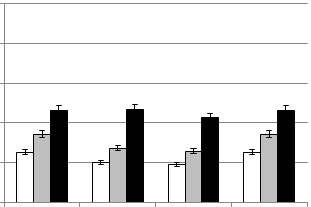

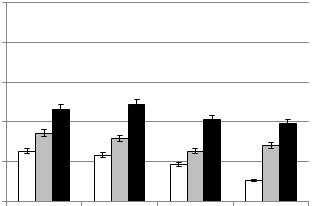


**Locus_6525 (HSP89.1)**

**5**

**4**

**3**

**2**

**1**

**0**


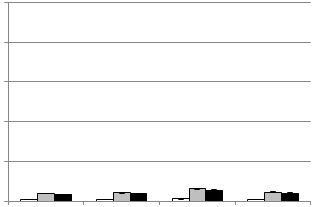

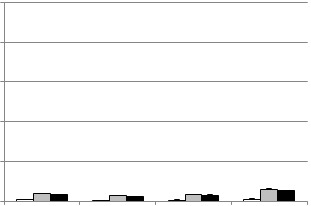

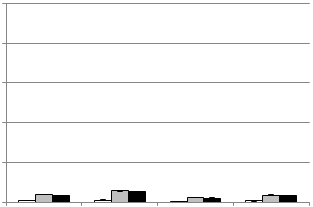


**Tip-burn resistant**


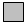


**Tip-burn susceptible**


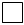


**Locus_6468 (HSP20)**

**5**

**4**

**3**

**2**

**1**

**0**

**Kale**


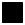


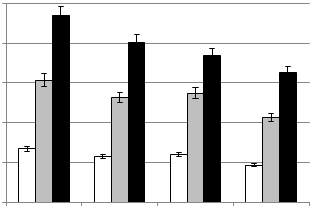

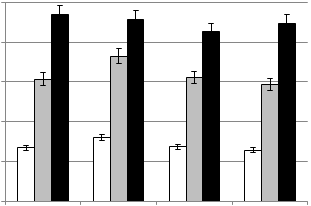

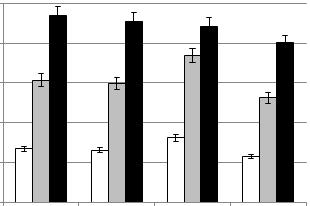


**Locus_16469 (DnaJ)**

**5**

**4**

**3**

**2**

**1**

**0**

**0 1 3 6 (h)**

**0 1 3 6 (h)**

**0 1 3 6 (h)**

**Figure S3.** Phenotypic appearance (A) and gene qRT-PCR results (B) of two cabbages (tip-burn susceptible and resistant lines) and kale under heat-shock stress, freezing-stress and drought stress conditions.
