# Supplementary material for: Intracellular Ca2+ and K+ concentration in Brassica oleracea leaf induces differential expression of transporter and stress-related genes
Source: BMC Genomics. 2016 Mar 9;17:211. doi: 10.1186/s12864-016-2512-x (PMC4784358; doi:10.1186/s12864-016-2512-x)
Supplement: Additional file 7: Figure S4. — Selected up- or down-regulated genes based on increasing Ca2+ and K+ concentration in the three lines with three different leaf positions. (DOCX 169 kb) [file 12864_2016_2512_MOESM7_ESM.docx]

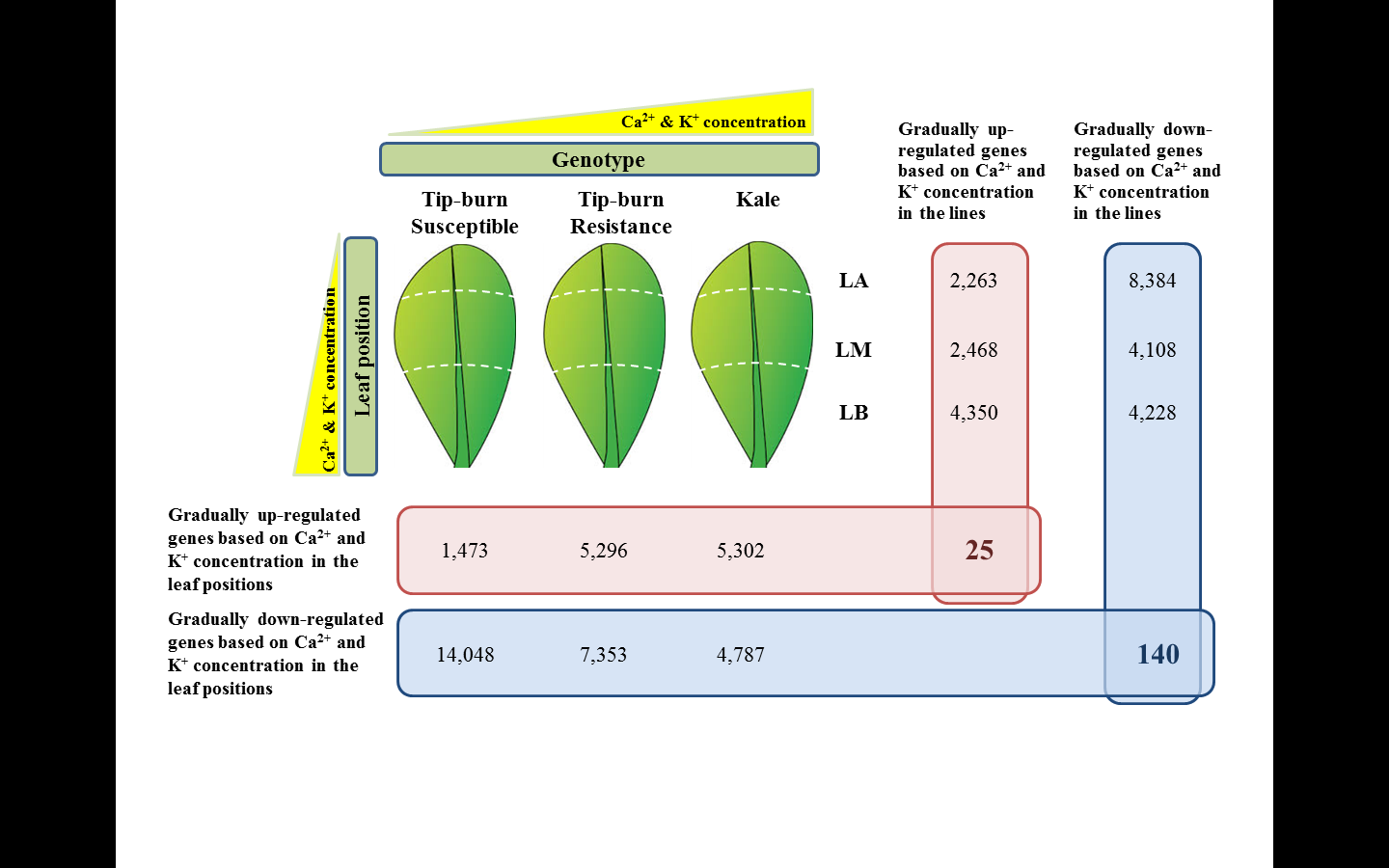


**Figure S4.** Selected up- or down-regulated genes based on increasing Ca^2+^ and K^+^ concentration in the three lines with three different leaf positions.
