# Supplementary material for: Intracellular Ca2+ and K+ concentration in Brassica oleracea leaf induces differential expression of transporter and stress-related genes
Source: BMC Genomics. 2016 Mar 9;17:211. doi: 10.1186/s12864-016-2512-x (PMC4784358; doi:10.1186/s12864-016-2512-x)
Supplement: Additional file 8: Table S4. — Transporter genes with significantly altered transcript levels. (DOCX 51 kb) [file 12864_2016_2512_MOESM8_ESM.docx]

**Table S4.** Transporter genes with significantly altered transcript levels.

| **ID** | **Gene family annotation** | **Tip-burn susceptible** | | | **Tip-burn resistant** | | | **Kale** | | |
| --- | --- | --- | --- | --- | --- | --- | --- | --- | --- | --- |
|  |  | **LA** | **LM** | **LB** | **LA** | **LM** | **LB** | **LA** | **LM** | **LB** |
| Locus_5828 | Lipid transporters | 1.00 | 3.16 | 3.44 | 4.17 | 4.28 | 4.70 | 4.63 | 4.72 | 4.29 |
| Locus_12006 | EXS (ERD1/XPR1/SYG1) family protein | 1.00 | 2.18 | 3.61 | 3.42 | 4.20 | 3.88 | 2.46 | 2.92 | 2.43 |
| Locus_631 | MATE efflux family protein | 1.00 | -0.74 | -0.30 | 10.11 | 9.99 | 9.98 | 9.51 | 9.52 | 9.48 |
| Locus_4034 | Aminopeptidase P1 | 1.00 | -0.48 | -0.52 | 7.46 | 7.50 | 7.35 | 8.15 | 8.11 | 8.20 |
| Locus_7025 | Catalytics | 1.00 | -0.48 | -0.52 | 7.18 | 6.33 | 6.26 | 7.17 | 7.01 | 7.20 |
| Locus_8783 | Vacuolar protein sorting-associated protein 28 | 1.00 | -0.48 | -0.52 | 7.05 | 6.96 | 6.86 | 6.76 | 6.77 | 6.82 |
| Locus_6186 | K^+^ efflux antiporter 1 | 1.00 | 0.52 | -1.10 | 3.79 | 4.10 | 4.82 | 6.52 | 6.58 | 6.32 |
| Locus_15412 | Rubredoxin-like superfamily protein | 1.00 | -0.48 | -0.52 | 5.73 | 6.09 | 5.92 | 6.34 | 6.58 | 6.23 |
| Locus_3325 | K^+^ efflux antiporter 1 | 1.00 | -0.48 | 0.07 | 5.67 | 5.89 | 6.14 | 6.33 | 6.11 | 6.04 |
| Locus_7205 | Golgi nucleotide sugar transporter 1 | 1.00 | -0.48 | 0.48 | 5.31 | 5.39 | 5.47 | 5.82 | 6.05 | 5.91 |
| Locus_24125 | Secretory carrier membrane protein (SCAMP) | 1.00 | -0.48 | -0.52 | 6.25 | 5.04 | 4.98 | 6.46 | 5.60 | 5.56 |
| Locus_8577 | Vesicle-associated membrane protein 713 | 1.00 | -1.80 | -1.84 | 5.25 | 5.13 | 5.19 | 5.29 | 5.10 | 5.12 |
| Locus_24656 | Heavy metal transport protein | 1.00 | -0.48 | -0.52 | 5.67 | 4.64 | 4.61 | 5.46 | 5.21 | 5.09 |
| Locus_40647 | ATPase, V1 complex, subunit B protein | 1.00 | -0.48 | -0.52 | 4.49 | 4.19 | 4.36 | 4.94 | 4.58 | 4.97 |
| Locus_13564 | Mitochondrial substrate carrier protein | 1.00 | 0.84 | 1.07 | 5.46 | 4.99 | 5.18 | 4.77 | 4.63 | 4.95 |
| Locus_4188 | Uurea transmembrane transporters | 1.00 | -0.48 | -0.52 | 5.21 | 5.51 | 5.85 | 3.63 | 4.87 | 4.77 |
| Locus_38199 | Sugar transporter protein 7 | 1.00 | 0.52 | 0.07 | 4.68 | 5.30 | 5.44 | 4.92 | 4.36 | 4.69 |
| Locus_3750 | Cyclic nucleotide gated channel 1 | 1.00 | -0.48 | 1.07 | 4.23 | 4.47 | 4.66 | 3.75 | 4.01 | 4.57 |
| Locus_43124 | Cyclic nucleotide-gated channel 14 | 1.00 | 1.11 | 1.07 | 2.71 | 3.19 | 3.34 | 3.42 | 3.68 | 4.51 |
| Locus_37346 | Xanthine/uracil permease protein | 1.00 | 0.11 | -0.52 | 3.17 | 3.25 | 3.16 | 3.63 | 4.45 | 4.37 |
| Locus_7776 | TLC ATP/ADP transporter | 1.00 | -0.48 | -0.52 | 5.17 | 5.04 | 5.34 | 4.27 | 4.08 | 4.34 |
| Locus_13051 | MATE efflux family protein | 1.00 | 1.26 | 1.80 | 4.05 | 4.71 | 5.05 | 3.99 | 3.89 | 4.25 |
| Locus_12462 | UDP-galactose transporter 3 | 1.00 | -0.23 | 0.24 | 4.00 | 3.72 | 3.74 | 4.45 | 4.13 | 4.20 |
| Locus_7401 | Casein kinase I-like 8 | 1.00 | 0.16 | 0.50 | 3.50 | 3.86 | 3.55 | 3.98 | 4.38 | 4.20 |
| Locus_39502 | Beta glucosidase 15 | 1.00 | -1.70 | 1.40 | 3.07 | 3.11 | 2.82 | 4.10 | 3.97 | 4.09 |
| Locus_16297 | ABC transporter family protein | 1.00 | -0.74 | 1.54 | 3.58 | 3.80 | 3.72 | 4.21 | 4.03 | 4.08 |
| Locus_3273 | NDH-dependent cyclic electron flow 1 | 1.00 | -0.48 | -0.52 | 5.72 | 6.16 | 6.17 | 4.32 | 4.21 | 4.02 |
| Locus_1355 | Multidrug resistance-associated protein 5 | 1.00 | 0.24 | 0.59 | 3.10 | 3.66 | 3.63 | 4.00 | 4.12 | 3.87 |
| Locus_4636 | Iron-regulated transporter 1 | 1.00 | -0.48 | -0.52 | 4.92 | 4.28 | 4.81 | 2.92 | 3.18 | 3.85 |
| Locus_2393 | ADP/ATP carrier 2 | 1.00 | 0.66 | 1.41 | 3.52 | 4.26 | 4.30 | 2.80 | 3.39 | 3.83 |
| Locus_1699 | Vaculolar sorting receptor 3 | 1.00 | -0.74 | -0.30 | 3.25 | 2.42 | 2.17 | 3.96 | 3.78 | 3.82 |
| Locus_1883 | Zinc induced facilitator 1 | 1.00 | -0.90 | -0.52 | 3.87 | 3.47 | 3.33 | 3.71 | 3.76 | 3.82 |
| Locus_15100 | Cation/H^+^ exchanger 19 | 1.00 | -0.48 | -0.52 | 3.61 | 4.09 | 3.72 | 3.16 | 3.11 | 3.75 |
| Locus_21340 | ATPase E1-E2 type family protein | 1.00 | -0.48 | -0.52 | 6.31 | 6.00 | 5.88 | 3.46 | 3.11 | 3.71 |
| Locus_45789 | Small and basic intrinsic protein 2;1 | 1.00 | -0.48 | 0.48 | 4.29 | 3.84 | 4.44 | 3.82 | 4.28 | 3.71 |
| Locus_4599 | Translocase inner membrane subunit 44-2 | 1.00 | 1.65 | 1.73 | 2.41 | 2.97 | 3.16 | 3.67 | 3.54 | 3.69 |
| Locus_39463 | Major facilitator superfamily protein | 1.00 | -0.16 | 1.70 | 2.82 | 3.09 | 3.13 | 3.26 | 3.49 | 3.63 |
| Locus_10464 | 1-amino-cyclopropane-1-carboxylate synthase 7 | 1.00 | 0.33 | 0.48 | 3.49 | 4.06 | 4.16 | 2.82 | 3.99 | 3.55 |
| Locus_15085 | Phosphate transporter 4;6 | 1.00 | -0.48 | -0.52 | 3.75 | 2.67 | 2.22 | 4.11 | 2.97 | 3.55 |
| Locus_45135 | Clathrin adaptor complexes medium subunit | 1.00 | -0.26 | 1.71 | 3.82 | 4.70 | 4.66 | 2.46 | 2.81 | 3.45 |
| Locus_7272 | TRICHOME BIREFRINGENCE-LIKE 27 | 1.00 | -0.48 | -0.52 | 3.84 | 3.06 | 2.96 | 3.46 | 3.37 | 3.42 |
| Locus_998 | Major facilitator superfamily protein | 1.00 | 1.08 | 1.20 | 3.38 | 3.25 | 3.26 | 3.35 | 3.11 | 3.34 |
| Locus_11387 | Rab escort protein | 1.00 | 0.52 | 0.48 | 3.56 | 4.02 | 3.72 | 4.16 | 3.58 | 3.32 |
| Locus_2135 | Mitochondrial ATP synthase subunit G protein | 1.00 | -0.10 | 0.28 | 3.12 | 3.22 | 2.95 | 2.94 | 2.81 | 3.23 |
| Locus_205 | clone eighty-four | 1.00 | 1.02 | 1.61 | 3.08 | 3.63 | 3.82 | 2.25 | 2.83 | 3.19 |
| Locus_4041 | Inorganic H pyrophosphatase family protein | 1.00 | 1.28 | 1.72 | 2.19 | 3.13 | 2.95 | 2.61 | 3.02 | 3.13 |
| Locus_38016 | MATE efflux family protein | 1.00 | -0.48 | -0.52 | 4.49 | 4.84 | 5.10 | 3.85 | 3.76 | 3.05 |
| Locus_8750 | Nodulin MtN21 /EamA-like transporter | 1.00 | -1.07 | -1.10 | 2.87 | 2.40 | 2.64 | 3.34 | 2.78 | 3.00 |
| Locus_27821 | Magnesium transporter 2 | 1.00 | 1.33 | 1.07 | 2.66 | 2.80 | 2.64 | 2.42 | 3.11 | 2.98 |
| Locus_9373 | SH3 domain of STAM 1 | 1.00 | 0.96 | 1.04 | 2.58 | 2.65 | 2.17 | 3.20 | 3.11 | 2.94 |
| Locus_2500 | Clathrin, heavy chain | 1.00 | 1.27 | 1.65 | 2.43 | 2.44 | 2.60 | 2.56 | 2.88 | 2.90 |

Table S4 (Continued).

| **ID** | **Gene family annotation** | **Tip-burn susceptible** | | | **Tip-burn resistant** | | | **Kale** | | |
| --- | --- | --- | --- | --- | --- | --- | --- | --- | --- | --- |
|  |  | **LA** | **LM** | **LB** | **LA** | **LM** | **LB** | **LA** | **LM** | **LB** |
| Locus_16053 | Glutamate receptor 3.4 | 1.00 | 0.81 | 0.65 | 2.21 | 2.61 | 2.66 | 3.15 | 2.80 | 2.79 |
| Locus_41051 | Sucrose transporter 2 | 1.00 | 0.40 | 0.13 | 3.13 | 3.14 | 3.27 | 2.75 | 2.95 | 2.85 |
| Locus_41010 | Sec14p-like phosphatidylinositol transfer protein | 1.00 | 1.02 | 1.80 | 5.09 | 4.20 | 4.47 | 3.07 | 3.40 | 2.78 |
| Locus_11094 | YELLOW STRIPE like 6 | 1.00 | -0.48 | -0.52 | 3.29 | 3.31 | 2.64 | 2.98 | 3.72 | 2.63 |
| Locus_27364 | FAD-binding Berberine family protein | 1.00 | -0.48 | -0.52 | 6.97 | 6.66 | 6.61 | 2.46 | 2.53 | 2.63 |
| Locus_1186 | RAB homolog 1 | 1.00 | -1.26 | -2.72 | 3.16 | 2.19 | 2.35 | 3.04 | 2.69 | 2.57 |
| Locus_13188 | Integral membrane HRF1 family protein | 1.00 | 0.52 | 1.07 | 3.51 | 2.67 | 2.81 | 2.78 | 2.81 | 2.55 |
| Locus_6982 | Cytochrome P450, family 71 subfamily B | 1.00 | 0.39 | 0.72 | 3.75 | 3.34 | 3.36 | 3.42 | 2.81 | 2.51 |
| Locus_21623 | Lipid transfer protein 3 | 1.00 | -0.36 | -0.97 | 2.64 | 2.23 | 2.24 | 2.29 | 2.18 | 2.48 |
| Locus_7596 | Chloride channel C | 1.00 | -0.03 | 0.08 | 2.56 | 2.43 | 2.28 | 2.57 | 2.52 | 2.46 |
| Locus_42921 | DNAse I-like superfamily protein | 1.00 | -1.80 | -1.52 | 2.05 | 2.45 | 2.77 | 2.25 | 2.28 | 2.39 |
| Locus_12333 | ABC2 homolog 12 | 1.00 | 0.78 | 0.80 | 2.73 | 2.56 | 2.47 | 2.58 | 2.48 | 2.36 |
| Locus_2157 | MATE efflux family protein | 1.00 | 0.51 | 1.09 | 2.63 | 2.33 | 2.47 | 2.08 | 2.21 | 2.26 |
| Locus_6201 | P-glycoprotein 9 | 1.00 | 0.59 | 1.43 | 2.12 | 2.77 | 2.79 | 2.88 | 2.74 | 2.25 |
| Locus_34895 | Endomembrane protein 70 protein family | 1.00 | -0.48 | -0.52 | -0.29 | -0.33 | -0.36 | 8.04 | 7.50 | 7.51 |
| Locus_7648 | Amino acid permease 8 | 1.00 | -0.48 | 0.07 | -0.29 | -0.33 | -0.36 | 5.23 | 5.53 | 5.73 |
| Locus_34169 | Major facilitator superfamily protein | 1.00 | -0.48 | -0.52 | -0.29 | -0.33 | -0.36 | 5.83 | 5.55 | 5.72 |
| Locus_33906 | Purine permease 8 | 1.00 | -0.48 | -0.52 | -0.29 | -0.33 | -0.36 | 5.86 | 5.86 | 5.44 |
| Locus_38616 | Intracellular transport protein | 1.00 | -0.48 | -0.52 | 0.70 | 0.66 | 0.22 | 2.85 | 4.28 | 5.26 |
| Locus_10016 | Golgi snare 12 | 1.00 | -0.08 | 0.89 | 1.54 | 1.82 | 1.96 | 5.18 | 5.78 | 5.15 |
| Locus_31191 | Galactose oxidase/kelch repeat protein | 1.00 | 1.01 | -0.03 | -0.03 | 0.93 | 1.90 | 2.78 | 4.19 | 4.71 |
| Locus_51263 | Myosin heavy chain-related | 1.00 | 0.41 | 0.48 | -0.81 | 0.04 | 0.40 | 3.38 | 3.92 | 4.67 |
| Locus_18788 | Ca^2+^ activated outward rectifying K^+^ channel 6 | 1.00 | -0.48 | -0.52 | -0.29 | 1.84 | 1.81 | 4.02 | 2.89 | 4.42 |
| Locus_44678 | S-adenosyl-L-methionine-dependent methyltransferases | 1.00 | 0.69 | 0.45 | 0.53 | -0.04 | 0.72 | 4.29 | 4.52 | 4.38 |
| Locus_32385 | TUDOR-SN protein 1 | 1.00 | -0.48 | -0.52 | -0.29 | -0.33 | -0.36 | 3.75 | 4.31 | 4.22 |
| Locus_5300 | Equilibrative nucleotide transporter 1 | 1.00 | 0.49 | 0.60 | -0.24 | 0.89 | 1.67 | 4.42 | 4.36 | 4.19 |
| Locus_52555 | Vacuolar protein sorting 45 | 1.00 | 1.11 | 0.48 | -0.29 | -0.33 | -0.36 | 4.22 | 4.60 | 4.13 |
| Locus_41913 | Major facilitator superfamily protein | 1.00 | -0.48 | 0.48 | -0.29 | -0.33 | -0.36 | 3.05 | 3.85 | 4.02 |
| Locus_15774 | Glutamate receptor 5 | 1.00 | 1.00 | 0.16 | 1.19 | 1.04 | 1.40 | 3.33 | 3.55 | 3.94 |
| Locus_30352 | Elicitor-activated gene 3-2 | 1.00 | -0.48 | -0.52 | -0.29 | -0.33 | -0.36 | 2.55 | 2.04 | 3.71 |
| Locus_21564 | Inositol transporter 1 | 1.00 | 0.85 | 1.39 | 0.52 | 0.48 | 1.81 | 2.75 | 3.28 | 3.69 |
| Locus_9217 | Nodulin MtN21/EamA-like transporter protein | 1.00 | 1.02 | 1.48 | -1.88 | 0.89 | 0.37 | 2.81 | 2.78 | 3.63 |
| Locus_9629 | Ca^2+^ activated outward rectifying K^+^ channel 5 | 1.00 | 1.52 | 1.70 | -0.88 | 1.08 | 1.05 | 3.66 | 4.26 | 3.52 |
| Locus_7712 | MD-2-related lipid recognition domain-containing protein | 1.00 | 0.60 | 0.76 | -0.35 | 0.11 | -0.17 | 3.28 | 3.57 | 3.51 |
| Locus_643 | Voltage dependent anion channel 1 | 1.00 | 0.74 | 0.53 | -1.31 | -0.57 | -0.90 | 2.68 | 3.17 | 3.45 |
| Locus_16643 | K^+^ efflux antiporter 4 | 1.00 | 0.44 | 0.27 | -0.92 | -1.22 | -1.57 | 2.39 | 2.76 | 3.29 |
| Locus_331 | emp24/gp25L/p24 family protein | 1.00 | -0.66 | -1.27 | -0.79 | -0.48 | -0.16 | 3.40 | 3.15 | 3.21 |
| Locus_20750 | Major facilitator superfamily protein | 1.00 | -2.39 | -3.42 | -2.20 | -3.24 | -3.27 | 2.00 | 2.06 | 3.18 |
| Locus_44892 | Clathrin adaptor complexes medium subunit | 1.00 | 0.92 | 0.67 | -0.67 | -1.05 | -0.93 | 2.21 | 2.88 | 3.16 |
| Locus_6946 | Nuclear transport factor 2 (NTF2) protein | 1.00 | -0.11 | 0.73 | 0.73 | -0.47 | -0.36 | 3.28 | 3.23 | 3.12 |
| Locus_38872 | ABC transporter family protein | 1.00 | 1.16 | 1.63 | -0.01 | 1.14 | 1.44 | 2.21 | 2.67 | 3.04 |
| Locus_27540 | Target SNARE coiled-coil domain protein | 1.00 | 0.78 | 1.41 | -1.62 | 0.35 | -0.36 | 3.05 | 3.49 | 2.97 |
| Locus_38540 | Galactose oxidase/kelch repeat protein | 1.00 | -1.59 | -0.63 | 0.18 | -0.71 | -0.48 | 2.06 | 2.82 | 2.94 |
| Locus_42050 | MATE efflux family protein | 1.00 | -1.48 | 1.07 | 1.51 | -0.33 | 0.22 | 2.42 | 2.11 | 2.92 |
| Locus_34370 | Nucleotide-sugar transporter family protein | 1.00 | -0.48 | -0.52 | 0.29 | 0.25 | 0.64 | 3.05 | 2.97 | 2.92 |
| Locus_38588 | High-affinity K^+^ transporter 1 | 1.00 | 0.85 | 1.64 | 1.56 | 1.38 | 1.87 | 3.04 | 2.68 | 2.75 |
| Locus_664 | Light harvesting complex photosystem II subunit 6; calcium ion transport | 1.00 | 0.98 | 1.80 | 1.61 | -0.01 | 0.96 | 3.01 | 3.33 | 2.71 |
| Locus_38253 | B-cell receptor-associated 31-like | 1.00 | 0.27 | 0.83 | 0.81 | 0.91 | 1.22 | 2.77 | 2.46 | 2.65 |

Table S4 (Continued).

| **ID** | **Gene family annotation** | **Tip-burn susceptible** | | | **Tip-burn resistant** | | | **Kale** | | |
| --- | --- | --- | --- | --- | --- | --- | --- | --- | --- | --- |
|  |  | **LA** | **LM** | **LB** | **LA** | **LM** | **LB** | **LA** | **LM** | **LB** |
| Locus_11073 | Plasma membrane intrinsic protein 1;4 | 1.00 | 0.80 | 1.19 | 0.22 | 0.69 | 1.08 | 2.76 | 2.54 | 2.56 |
| Locus_12206 | Peptide transporter 2 | 1.00 | -0.48 | 0.48 | 1.03 | 1.99 | -0.36 | 2.71 | 2.18 | 2.63 |
| Locus_3895 | Glutamate receptor 5 | 1.00 | 0.67 | 0.91 | -1.01 | -0.36 | -0.63 | 3.02 | 2.65 | 2.63 |
| Locus_14935 | Riboflavin synthase-like protein | 1.00 | -0.48 | -0.52 | -0.29 | -0.33 | -0.36 | 2.55 | 2.72 | 2.46 |
| Locus_14675 | ABC-2 type transporter protein | 1.00 | 1.16 | 1.29 | 1.17 | 1.10 | 1.00 | 2.23 | 2.22 | 2.43 |
| Locus_52393 | ALA-interacting subunit 5 | 1.00 | -0.57 | -0.82 | -0.71 | -1.33 | -0.90 | 2.74 | 2.85 | 2.39 |
| Locus_18147 | Nodulin MtN21/EamA-like transporter protein | 1.00 | 0.16 | 1.77 | -0.14 | 1.45 | 1.27 | 2.75 | 2.98 | 2.34 |
| Locus_14291 | Galactose oxidase/kelch repeat protein | 1.00 | 1.19 | 1.74 | 0.52 | 0.51 | 0.61 | 2.17 | 2.06 | 2.29 |
| Locus_5048 | Nitrate transmembrane transporters | 1.00 | 0.68 | 1.39 | 0.94 | 1.36 | 1.52 | 2.02 | 2.12 | 2.28 |
| Locus_5956 | Aluminium activated malate transporter | 1.00 | 0.80 | 1.43 | 1.26 | 1.40 | 1.42 | 2.02 | 2.18 | 2.25 |
| Locus_38107 | D6 protein kinase | 1.00 | 0.80 | 1.49 | 1.37 | 1.44 | 1.55 | 2.16 | 2.29 | 2.25 |
| Locus_6964 | EXS (ERD1/XPR1/SYG1) protein; phoshpate ion transport | 1.00 | 0.47 | 1.03 | 1.53 | 1.69 | 1.43 | 2.40 | 2.54 | 2.24 |
| Locus_9044 | Manganese tracking factor | 1.00 | 0.48 | 0.91 | 0.46 | 0.06 | 0.03 | 2.49 | 2.38 | 2.21 |
| Locus_8735 | Ubiquitin-like superfamily protein | 1.00 | 1.15 | 1.53 | 1.43 | 1.63 | 1.95 | 2.33 | 2.25 | 2.19 |
| Locus_23286 | MATE efflux family protein | 1.00 | 0.80 | 1.68 | 0.58 | 0.77 | 0.84 | 2.14 | 2.16 | 2.16 |
| Locus_27869 | YELLOW STRIPE like 2 | 1.00 | 1.13 | 1.65 | 1.44 | 1.62 | 1.26 | 2.59 | 2.50 | 2.16 |
| Locus_41609 | Polyketide cyclase/lipid transport protein | 1.00 | 0.01 | 0.97 | 1.47 | 1.51 | 0.90 | 2.39 | 3.02 | 2.05 |
| Locus_986 | Got1/Sft2-like vescicle transport protein | 1.00 | 0.93 | 1.36 | 1.12 | 0.08 | 1.22 | 2.04 | 2.14 | 2.05 |
| Locus_30774 | MATE efflux family protein | 1.00 | -0.48 | -0.52 | -0.29 | 0.25 | -0.36 | 3.22 | 2.53 | 2.05 |
