# Supplementary material for: Intracellular Ca2+ and K+ concentration in Brassica oleracea leaf induces differential expression of transporter and stress-related genes
Source: BMC Genomics. 2016 Mar 9;17:211. doi: 10.1186/s12864-016-2512-x (PMC4784358; doi:10.1186/s12864-016-2512-x)
Supplement: Additional file 9: Table S5. — Response to stress genes with significantly altered transcript levels. (DOCX 51 kb) [file 12864_2016_2512_MOESM9_ESM.docx]

**Table S5**. Response to stress genes with significantly altered transcript levels.

| **ID** | **Gene family annotation** | **Tip-burn susceptible** | | | **Tip-burn resistant** | | | **Kale** | | |
| --- | --- | --- | --- | --- | --- | --- | --- | --- | --- | --- |
|  |  | **LA** | **LM** | **LB** | **LA** | **LM** | **LB** | **LA** | **LM** | **LB** |
| Locus_9065 | Leucine-rich repeat (LRR) family protein | 1.00 | 2.61 | 3.18 | 2.41 | 3.37 | 4.44 | 3.42 | 3.68 | 4.08 |
| Locus_16469 | Chaperone DnaJ-domain superfamily protein | 1.00 | 2.16 | 2.94 | 2.51 | 2.42 | 2.28 | 3.48 | 2.97 | 3.75 |
| Locus_49615 | Pathogenesis-related thaumatin protein | 1.00 | 2.65 | 2.32 | 4.11 | 3.89 | 4.25 | 3.77 | 3.77 | 3.42 |
| Locus_3698 | Eukaryotic translation initiation factor 4B1 | 1.00 | 2.61 | 3.29 | 2.88 | 2.25 | 3.59 | 3.75 | 4.11 | 3.32 |
| Locus_629 | Adenine nucleotide alpha hydrolases-like protein | 1.00 | -0.48 | -0.52 | 8.40 | 8.63 | 8.49 | 9.28 | 8.66 | 8.87 |
| Locus_9400 | Low temperature and salt responsive protein family | 1.00 | -0.48 | -0.52 | 7.29 | 7.62 | 7.65 | 8.26 | 8.75 | 8.58 |
| Locus_9666 | Lipase/lipooxygenase, PLAT/LH2 family protein | 1.00 | 0.10 | -1.52 | 4.10 | 3.82 | 4.27 | 7.84 | 7.75 | 8.01 |
| Locus_37911 | Disease resistance protein (TIR-NBS-LRR class) | 1.00 | -0.48 | -0.52 | 6.36 | 5.86 | 5.97 | 7.27 | 7.13 | 7.19 |
| Locus_12407 | Nucleoside-triphosphatases | 1.00 | 0.84 | 1.23 | 6.17 | 6.01 | 5.97 | 6.45 | 6.52 | 6.57 |
| Locus_9286 | Vacuolar protein sorting 41 | 1.00 | -0.48 | -0.52 | 6.90 | 6.21 | 6.64 | 6.33 | 6.19 | 5.93 |
| Locus_4050 | Mitochondrial HSO70 2 | 1.00 | -0.48 | -0.52 | 7.28 | 7.56 | 7.49 | 6.01 | 5.71 | 5.70 |
| Locus_8838 | Chalcone-flavanone isomerase protein | 1.00 | 0.52 | 0.48 | 5.78 | 5.60 | 5.10 | 6.34 | 6.17 | 5.59 |
| Locus_7737 | Pathogenesis-related thaumatin protein | 1.00 | -0.48 | -0.52 | 4.29 | 4.60 | 4.52 | 5.62 | 4.97 | 5.53 |
| Locus_40512 | Heat acclimation related protein | 1.00 | -0.48 | -0.52 | 5.25 | 4.99 | 5.38 | 5.05 | 4.25 | 4.65 |
| Locus_9057 | Peroxidase superfamily protein | 1.00 | -0.48 | -0.52 | 5.88 | 5.95 | 5.98 | 5.03 | 4.21 | 4.48 |
| Locus_24131 | Disease resistance family protein / LRR protein | 1.00 | -0.48 | -0.52 | 4.75 | 4.37 | 4.16 | 3.75 | 4.83 | 4.32 |
| Locus_6468 | HSP20-like chaperones superfamily protein | 1.00 | -0.48 | -0.52 | 5.08 | 5.21 | 5.28 | 4.85 | 4.87 | 4.32 |
| Locus_25540 | Mitochondrial import inner membrane translocase subunit Tim17/Tim22/Tim23 family protein | 1.00 | -0.48 | -0.52 | 3.95 | 3.71 | 4.49 | 4.44 | 4.15 | 4.29 |
| Locus_28270 | Alpha/beta-Hydrolases superfamily protein | 1.00 | -0.48 | -0.52 | 2.29 | 2.13 | 2.22 | 3.32 | 4.01 | 4.22 |
| Locus_17551 | AIG2-like (avirulence induced gene) protein | 1.00 | -0.48 | -0.52 | 3.99 | 2.99 | 2.96 | 4.48 | 4.15 | 4.19 |
| Locus_10758 | BTB-POZ and MATH domain 1 | 1.00 | -0.48 | 0.48 | 6.30 | 6.10 | 6.12 | 5.16 | 4.81 | 4.11 |
| Locus_24020 | HSP20-like chaperones superfamily protein | 1.00 | 0.11 | 1.29 | 4.82 | 4.63 | 4.43 | 4.31 | 3.95 | 4.08 |
| Locus_12442 | Seven transmembrane MLO family protein | 1.00 | -0.48 | -0.52 | 4.88 | 5.11 | 5.24 | 3.63 | 3.93 | 4.05 |
| Locus_38822 | ADR1-like 1 | 1.00 | 1.14 | 1.19 | 3.07 | 3.64 | 3.58 | 2.65 | 3.52 | 3.66 |
| Locus_38 | Copper/zinc superoxide dismutase 1 | 1.00 | 0.20 | 1.97 | 3.86 | 4.59 | 4.86 | 2.69 | 3.27 | 3.63 |
| Locus_4148 | DNAJ heat shock family protein | 1.00 | -0.48 | -0.52 | 3.03 | 3.62 | 3.77 | 2.92 | 2.72 | 3.37 |
| Locus_10454 | Disease resistance protein (CC-NBS-LRR class) | 1.00 | 1.37 | 1.85 | 2.80 | 2.62 | 2.78 | 3.24 | 3.56 | 3.28 |
| Locus_8135 | NDR1/HIN1-like 2 | 1.00 | -0.16 | 0.18 | 2.38 | 2.50 | 2.19 | 3.18 | 3.26 | 3.12 |
| Locus_1235 | NADH-dependent glutamate synthase 1 | 1.00 | 0.37 | 0.71 | 2.59 | 3.38 | 3.63 | 3.07 | 2.94 | 3.03 |
| Locus_42199 | Thioglucoside glucohydrolase 1 | 1.00 | -0.48 | -0.52 | 3.71 | 4.02 | 3.54 | 2.46 | 2.72 | 2.92 |
| Locus_23610 | Phytochrome-associated protein phosphatase type 2C | 1.00 | 0.19 | 1.58 | 2.89 | 2.86 | 2.54 | 2.83 | 2.48 | 2.80 |
| Locus_8611 | LRR and NB-ARC domains-containing Disease resistance protein | 1.00 | 1.25 | 1.67 | 2.38 | 2.82 | 2.58 | 2.87 | 2.16 | 2.65 |
| Locus_14437 | TBP-associated factor 4 | 1.00 | 0.87 | 1.63 | 3.03 | 3.01 | 3.12 | 2.52 | 2.53 | 2.65 |
| Locus_5605 | Inducer of photoxidative stress | 1.00 | -0.28 | 0.80 | 2.10 | 2.59 | 2.36 | 2.98 | 2.77 | 2.60 |
| Locus_14426 | Ascorbate peroxidase 2 | 1.00 | -0.48 | -0.52 | 4.10 | 3.67 | 3.34 | 2.63 | 2.89 | 2.55 |
| Locus_3593 | MLP-like protein 28 | 1.00 | 0.86 | 0.64 | 6.62 | 6.16 | 6.00 | 3.00 | 2.66 | 2.51 |
| Locus_744 | Ferredoxin-NADP(+)-oxidoreductase 2 | 1.00 | 0.03 | 0.21 | 2.34 | 2.41 | 2.34 | 2.68 | 2.46 | 2.45 |
| Locus_38108 | 4-(cytidine 5'-phospho)-2-C-methyl-D-erithritol kinase | 1.00 | 1.02 | 1.78 | 2.28 | 2.28 | 2.24 | 2.64 | 2.54 | 2.30 |
| Locus_4252 | Xanthine dehydrogenase 1 | 1.00 | 1.09 | 1.34 | 2.74 | 2.75 | 2.69 | 2.39 | 2.19 | 2.27 |
| Locus_45591 | Homolog of yeast autophagy 18 (ATG18) F | 1.00 | 1.50 | 1.93 | 2.23 | 2.39 | 2.10 | 2.27 | 2.15 | 2.26 |
| Locus_39914 | Lung seven transmembrane receptor protein | 1.00 | 0.77 | 1.38 | 3.18 | 2.94 | 2.97 | 2.43 | 2.12 | 2.22 |
| Locus_1153 | Peroxidase superfamily protein | 1.00 | 0.29 | 0.21 | 4.61 | 3.95 | 3.88 | 2.11 | 2.05 | 2.16 |
| Locus_8981 | S-adenosyl-L-methionine-dependent methyltransferases superfamily protein | 1.00 | 0.97 | 1.71 | 2.33 | 2.72 | 2.68 | 2.30 | 2.04 | 2.13 |
| Locus_16274 | NAD(P)-binding Rossmann-fold protein | 1.00 | 1.26 | 1.71 | 3.12 | 3.54 | 3.30 | 2.46 | 2.72 | 2.05 |
| Locus_21191 | Low temperature and salt responsive protein | 1.00 | -0.16 | 0.18 | -0.49 | 0.47 | 0.34 | 6.06 | 6.28 | 6.92 |
| Locus_38647 | Peroxidase superfamily protein | 1.00 | -0.14 | -0.48 | -4.65 | -2.11 | -2.40 | 4.36 | 4.94 | 5.68 |
| Locus_22894 | GAST1 protein homolog 5 | 1.00 | 1.36 | 1.03 | 0.12 | -0.11 | 0.51 | 4.95 | 5.03 | 5.31 |

Table S5 (Continued).

| **ID** | **Gene family annotation** | **Tip-burn susceptible** | | | **Tip-burn resistant** | | | **Kale** | | |
| --- | --- | --- | --- | --- | --- | --- | --- | --- | --- | --- |
|  |  | **LA** | **LM** | **LB** | **LA** | **LM** | **LB** | **LA** | **LM** | **LB** |
| Locus_17689 | Cytochrome P450, family 71, subfamily A, polypeptide 12 | 1.00 | 1.58 | 1.90 | -1.88 | -1.33 | -1.95 | 3.30 | 4.07 | 4.25 |
| Locus_33973 | Disease resistance protein (TIR-NBS-LRR class) | 1.00 | -0.48 | 0.07 | -0.29 | 0.25 | 0.22 | 5.26 | 5.08 | 4.75 |
| Locus_51499 | DRE-binding protein 2A | 1.00 | -0.48 | -0.52 | -0.29 | -0.33 | -0.36 | 3.37 | 4.31 | 4.46 |
| Locus_52524 | Disease resistance protein (TIR-NBS-LRR class) | 1.00 | -0.48 | -0.52 | 0.29 | -0.33 | -0.36 | 4.98 | 5.13 | 4.22 |
| Locus_41805 | Disease resistance protein (TIR-NBS-LRR class) | 1.00 | 0.88 | 1.63 | 0.54 | 0.44 | -0.59 | 3.15 | 3.82 | 4.17 |
| Locus_45319 | S-adenosyl-L-methionine-dependent methyltransferases superfamily protein | 1.00 | -0.48 | -0.52 | -0.29 | 0.25 | 0.22 | 2.37 | 2.63 | 4.16 |
| Locus_32055 | Beta-1,3-glucanase 2 | 1.00 | -0.48 | -0.52 | -0.29 | -0.33 | -0.36 | 2.55 | 2.18 | 4.05 |
| Locus_33912 | Disease resistance protein (TIR-NBS-LRR class) | 1.00 | 0.10 | 0.49 | -0.56 | -0.11 | -0.95 | 3.87 | 4.00 | 3.95 |
| Locus_11419 | Embryo-specific protein 3 (ATS3) | 1.00 | -0.62 | 0.98 | 0.64 | 1.34 | 1.39 | 2.87 | 2.92 | 3.93 |
| Locus_27787 | AFP2 (ABI five-binding protein 2) family protein | 1.00 | 1.22 | 0.11 | -2.39 | -2.03 | -2.81 | 3.20 | 3.59 | 3.89 |
| Locus_52770 | Peroxidase superfamily protein | 1.00 | -0.48 | -0.52 | -0.29 | -0.33 | -0.36 | 4.51 | 4.31 | 3.85 |
| Locus_6525 | Heat shock protein 89.1 | 1.00 | 0.30 | 1.54 | 0.81 | 1.47 | 1.36 | 3.56 | 3.90 | 3.82 |
| Locus_5197 | KPC1 related protein | 1.00 | -0.48 | 0.07 | -0.29 | 0.67 | 1.64 | 4.24 | 5.25 | 3.71 |
| Locus_38054 | Zinc finger C-x8-C-x5-C-x3-H type protein | 1.00 | 0.46 | 0.64 | -0.29 | 0.49 | 0.48 | 2.50 | 3.31 | 3.47 |
| Locus_11913 | Cytosolic invertase 1 | 1.00 | 0.11 | 1.94 | 1.29 | 1.25 | 1.81 | 2.71 | 2.81 | 3.42 |
| Locus_40698 | Disease resistance protein (TIR-NBS-LRR class) | 1.00 | 0.52 | 1.29 | 1.17 | 1.25 | 1.34 | 2.89 | 3.21 | 3.41 |
| Locus_38086 | 3-ketoacyl-CoA synthase 16 | 1.00 | 0.81 | 1.11 | 0.54 | 0.62 | 0.53 | 3.58 | 3.37 | 3.38 |
| Locus_1906 | TPR-like protein | 1.00 | 0.81 | 1.77 | 1.28 | 1.75 | 1.86 | 3.08 | 3.34 | 3.26 |
| Locus_1135 | Chaperone protein htpG family protein | 1.00 | -0.41 | 0.13 | 1.35 | 1.56 | 1.53 | 2.80 | 3.13 | 3.21 |
| Locus_881 | Heat shock cognate protein 70-1 | 1.00 | 0.61 | 0.68 | 1.20 | 1.76 | 1.85 | 2.77 | 3.10 | 3.06 |
| Locus_47544 | Far-red impaired responsive (FAR1) protein | 1.00 | -0.48 | 0.38 | 1.48 | 1.32 | 0.83 | 3.37 | 3.00 | 3.01 |
| Locus_16953 | ORMDL family protein | 1.00 | 0.30 | 0.44 | -0.45 | 0.36 | 0.58 | 3.12 | 3.16 | 3.00 |
| Locus_40721 | Receptor like protein 12 | 1.00 | 0.34 | 1.28 | 1.40 | 1.90 | 1.75 | 2.34 | 2.97 | 2.99 |
| Locus_3770 | Alpha/beta-Hydrolases superfamily protein | 1.00 | 1.45 | 1.95 | 0.71 | 0.96 | 1.18 | 2.51 | 3.17 | 2.94 |
| Locus_27546 | Disease resistance protein (CC-NBS-LRR class) | 1.00 | 1.04 | 1.99 | 0.73 | 1.10 | 1.16 | 2.91 | 2.95 | 2.92 |
| Locus_15945 | Heat shock factor binding protein | 1.00 | 0.64 | -0.30 | 1.12 | -0.92 | 1.37 | 2.66 | 2.49 | 2.92 |
| Locus_23620 | Phytosulfokin receptor 1 | 1.00 | 0.59 | -0.33 | -0.75 | 0.29 | 0.18 | 2.90 | 2.79 | 2.88 |
| Locus_18753 | DA1-related protein 4 | 1.00 | 0.37 | 0.20 | -1.31 | -0.69 | -0.76 | 2.82 | 2.98 | 2.87 |
| Locus_2352 | BURP domain-containing protein | 1.00 | 0.59 | 1.16 | 0.97 | 1.02 | 0.96 | 2.36 | 2.68 | 2.84 |
| Locus_2877 | FAR1-related sequence 4 | 1.00 | 0.63 | 1.64 | 0.96 | 0.93 | 0.92 | 3.06 | 2.80 | 2.82 |
| Locus_27669 | Terpene synthase 04 | 1.00 | 1.04 | 1.24 | 1.98 | 1.92 | 1.90 | 2.94 | 2.73 | 2.77 |
| Locus_3020 | Oxophytodienoate-reductase 3 | 1.00 | 0.18 | 1.35 | 1.23 | 1.95 | 1.62 | 2.01 | 2.70 | 2.74 |
| Locus_15334 | BTB-POZ and MATH domain 1 | 1.00 | 0.09 | 0.88 | -1.82 | -0.61 | -0.53 | 2.68 | 2.64 | 2.72 |
| Locus_46048 | N-terminal nucleophile aminohydrolases (Ntn hydrolases) protein | 1.00 | -0.48 | -0.52 | -0.29 | -0.33 | -0.36 | 3.78 | 2.81 | 2.71 |
| Locus_17113 | Eukaryotic aspartyl protease family protein | 1.00 | -0.48 | -0.52 | 0.29 | -0.33 | 0.22 | 2.71 | 2.81 | 2.63 |
| Locus_34061 | NB-ARC domain-containing Disease resistance protein | 1.00 | 0.11 | -0.52 | -0.29 | -0.33 | -0.36 | 2.55 | 2.53 | 2.63 |
| Locus_15807 | Disease resistance protein (TIR-NBS-LRR class) | 1.00 | 0.52 | -0.52 | -0.88 | -0.92 | 1.30 | 2.81 | 2.42 | 2.60 |
| Locus_46186 | Disease resistance protein (TIR-NBS-LRR class) | 1.00 | 0.30 | 0.63 | 0.99 | 1.22 | 1.35 | 2.31 | 2.22 | 2.57 |
| Locus_43015 | Disease resistance protein (TIR-NBS-LRR class) | 1.00 | 0.84 | 1.44 | -1.71 | 0.37 | -0.78 | 2.27 | 3.28 | 2.53 |
| Locus_12189 | Insulinase (Peptidase family M16) protein | 1.00 | 0.67 | 0.48 | 0.12 | 0.40 | 0.99 | 3.20 | 2.72 | 2.48 |
| Locus_38576 | UDP-D-glucuronate 4-epimerase 5 | 1.00 | 0.42 | 1.47 | -1.17 | -0.47 | -0.65 | 2.59 | 2.16 | 2.45 |
| Locus_802 | Heat shock protein 81-2 | 1.00 | -0.53 | 0.46 | 0.89 | 1.24 | 1.24 | 2.08 | 2.51 | 2.45 |
| Locus_11853 | Disease resistance protein (TIR-NBS-LRR class) | 1.00 | 1.11 | 0.48 | 1.12 | 0.66 | 0.37 | 2.40 | 3.09 | 2.40 |
| Locus_12843 | Flavin-binding monooxygenase family protein | 1.00 | 1.04 | 1.87 | -0.43 | 0.88 | 0.46 | 2.55 | 2.39 | 2.37 |
| Locus_37937 | Isopropylmalate dehydrogenase 1 | 1.00 | 0.34 | -0.55 | 1.35 | 1.51 | 1.57 | 2.56 | 2.62 | 2.33 |
| Locus_7175 | Peroxidase 2 | 1.00 | 0.55 | 0.65 | 1.02 | 0.84 | 0.86 | 2.51 | 2.43 | 2.29 |
| Locus_15494 | Pyridoxine biosynthesis 1.2 | 1.00 | 0.61 | 1.65 | 0.19 | 0.43 | 0.22 | 2.17 | 2.06 | 2.25 |
| Locus_11450 | Purple acid phosphatase 10 | 1.00 | 0.44 | 0.58 | 0.20 | 0.44 | 0.23 | 2.20 | 2.15 | 2.22 |

Supplementary Table 5 (Continued).

| **ID** | **Gene family annotation** | **Tip-burn susceptible** | | | **Tip-burn resistant** | | | **Kale** | | |
| --- | --- | --- | --- | --- | --- | --- | --- | --- | --- | --- |
|  |  | **LA** | **LM** | **LB** | **LA** | **LM** | **LB** | **LA** | **LM** | **LB** |
| Locus_1690 | Arginine decarboxylase 2 | 1.00 | 0.62 | 0.69 | 0.03 | 1.02 | 1.47 | 2.00 | 2.06 | 2.16 |
| Locus_21917 | P-loop containing nucleoside triphosphate hydrolases superfamily protein | 1.00 | 0.27 | 0.98 | 1.94 | 1.84 | 1.71 | 2.45 | 2.14 | 2.18 |
| Locus_22264 | Cold regulated 15b | 1.00 | -0.48 | -0.52 | -0.29 | -0.33 | -0.36 | 3.42 | 2.81 | 2.16 |
| Locus_9315 | TPR-like protein | 1.00 | 0.68 | 1.12 | 1.72 | 1.88 | 1.95 | 2.38 | 2.29 | 2.14 |
| Locus_2079 | RCC1 protein | 1.00 | 0.48 | 1.61 | 1.51 | 1.80 | 1.60 | 2.05 | 2.11 | 2.12 |
| Locus_2753 | 3-deoxy-D-arabino-heptulosonate 7-phosphate synthase 1 | 1.00 | 0.77 | 1.20 | 1.03 | 1.16 | 1.34 | 2.18 | 2.19 | 2.06 |
| Locus_4756 | Mannose-binding lectin protein | 1.00 | 0.95 | 1.56 | 0.71 | 1.15 | 1.23 | 2.05 | 2.42 | 2.05 |
| Locus_469 | MLP-like protein 423 | 1.00 | 0.06 | -0.26 | -0.95 | -0.49 | -0.22 | 2.13 | 2.21 | 2.04 |
| Locus_35553 | Disease resistance protein (TIR-NBS-LRR class) | 1.00 | -0.48 | 0.07 | -0.29 | -0.33 | 0.22 | 2.78 | 2.72 | 2.04 |
| Locus_23590 | WD-40 repeat family protein | 1.00 | 1.33 | 1.66 | 0.03 | 0.25 | 0.42 | 2.10 | 2.06 | 2.03 |
